# Mammalian BTBD12 (SLX4) Protects against Genomic Instability during Mammalian Spermatogenesis

**DOI:** 10.1371/journal.pgen.1002094

**Published:** 2011-06-02

**Authors:** J. Kim Holloway, Swapna Mohan, Gabriel Balmus, Xianfei Sun, Andrew Modzelewski, Peter L. Borst, Raimundo Freire, Robert S. Weiss, Paula E. Cohen

**Affiliations:** 1 Department of Biomedical Sciences, College of Veterinary Medicine, Cornell University, Ithaca, New York, United States of America; 2 Unidad de Investigacion, Hospital Universitario de Canarias, Tenerife, Spain; Stowers Institute for Medical Research, United States of America

## Abstract

The mammalian ortholog of yeast Slx4, BTBD12, is an ATM substrate that functions as a scaffold for various DNA repair activities. Mutations of human *BTBD12* have been reported in a new sub-type of Fanconi anemia patients. Recent studies have implicated the fly and worm orthologs, MUS312 and HIM-18, in the regulation of meiotic crossovers arising from double-strand break (DSB) initiating events and also in genome stability prior to meiosis. Using a *Btbd12* mutant mouse, we analyzed the role of BTBD12 in mammalian gametogenesis. BTBD12 localizes to pre-meiotic spermatogonia and to meiotic spermatocytes in wildtype males. *Btbd12* mutant mice have less than 15% normal spermatozoa and are subfertile. Loss of BTBD12 during embryogenesis results in impaired primordial germ cell proliferation and increased apoptosis, which reduces the spermatogonial pool in the early postnatal testis. During prophase I, DSBs initiate normally in *Btbd12* mutant animals. However, DSB repair is delayed or impeded, resulting in persistent γH2AX and RAD51, and the choice of repair pathway may be altered, resulting in elevated MLH1/MLH3 focus numbers at pachynema. The result is an increase in apoptosis through prophase I and beyond. Unlike yeast Slx4, therefore, BTBD12 appears to function in meiotic prophase I, possibly during the recombination events that lead to the production of crossovers. In line with its expected regulation by ATM kinase, BTBD12 protein is reduced in the testis of *Atm^−/−^* males, and *Btbd12* mutant mice exhibit increased genomic instability in the form of elevated blood cell micronucleus formation similar to that seen in *Atm^−/−^* males. Taken together, these data indicate that BTBD12 functions throughout gametogenesis to maintain genome stability, possibly by co-ordinating repair processes and/or by linking DNA repair events to the cell cycle via ATM.

## Author Summary

Mutations in genes essential for genome maintenance during meiosis can result in severe disruptions to spermatogenesis and subsequent low fertility and/or birth defects in mammals. The mammalian homolog of yeast *Slx4*, BTBD12, plays a critical role in somatic cell repair in mice. Here, we show that this critical function extends to mammalian germ cells, by examining the effects of a *Btbd12* gene disruption in mice. *Btbd12* mutant mice show severely reduced fertility, as a result of both pre-meiotic spermatogonial proliferation defects and impairment of proper meiotic progression. BTBD12 appears to be required for normal progression of double-strand break repair events that result in the formation of crossovers between maternal and paternal homologous chromosomes, with *Btbd12* mutants displaying an increase in unrepaired breaks, impaired homologous chromosome interactions, and a slight increase in the number of crossover intermediates. BTBD12 protein is also down-regulated in the testes of *Atm* null mice, supporting previous studies showing that BTBD12 is a target of ATM kinase. These data provide new evidence about the role of BTBD12 in mammalian gametogenesis and are critical to furthering the understanding of the molecular processes involved in meiotic DNA repair.

## Introduction

*SLX1* and *SLX4* were identified, together with *MUS81* and *MMS4* (*Eme1* in mammals), in a *S. cerevisiae* screen for genes required for the viability of *sgs1*-deficient cells [1]. Slx1 is the founding member of a family of proteins with a predicted URI nuclease domain whose activity is enhanced 500-fold by its interaction with Slx4 [2]. Slx4 can also form complexes with Rad1-Rad10 [3], [4] to effect DSB repair during single-strand annealing in yeast [3], [5]. However, Slx4 can also act independently of both Slx1 and Rad1-Rad10 [2], [6], and is phosphorylated by Mec1 and Tel1, the yeast orthologs of ATR and ATM, respectively, in response to DNA damage [4].

Human, *C. elegans* and *D. melanogaster* orthologs of *SLX4* were described recently [7]–[10] and named BTBD12 (for BTB domain-containing protein-12), *Him-18*, and *Mus312*, respectively. These proteins are considerably diverged from their yeast counterpart; *BTBD12* encodes a 1834 amino acid protein, approximately 2.5-times larger than the yeast protein, and resembles its lower eukaryotic orthologs mostly in its C-terminal SAP and CCD domains [7]. Like the yeast ortholog, the human protein is a substrate of the ATM/ATR kinases [11] and its depletion also results in DNA damage sensitivity [8]. Recently, a subset of Fanconi anemia (FA) patients were found to have biallelic mutations in *BTBD12*, making this gene a novel complementation group for this disorder [12]. A complex of BTBD12 and SLX1 displays robust Holliday Junction (HJ) resolvase and 5′ flap endonuclease activity *in vitro*, and mammalian BTBD12 also binds to, and enhances the activity of, several DNA repair proteins including MUS81 [7], [10] and the MSH2-MSH3 heterodimer of the DNA mismatch repair (MMR) family [8], suggesting a role for this protein as a docking platform for structure-specific endonucleases.

Recent reports in *D. melanogaster* and *C. elegans* indicate that the *Btbd12* orthologs, *Mus312* and *Him-18*, respectively are essential for normal meiotic progression [13], [14]. In addition, *Him-18* appears to function pre-meiotically in the germ line, being required for repair at stalled replication forks [13], suggesting that Him-18 functions throughout germ cell development to maintain genomic integrity. Given these data, the primary goal of the current studies was to understand the function of BTBD12 in the germ line of mice, with the hypothesis that BTBD12 may be critical for the processing of homologous recombination intermediates, whether as the result of replication errors during pre-meiotic proliferation, or during the repair of double strand breaks (DSBs) that underlie meiotic recombination. For the latter, our studies were aimed at investigating the role of BTBD12 in the regulation of meiotic recombination events during prophase I in mammals, particularly those that ensure accurate segregation of maternal and paternal chromosomes at the first meiotic division. Principal amongst these is the formation of DNA crossovers between the homologs, as initiated by DSB induction through the activity of the SPO11 endonuclease [15], [16]. The resolution of DSBs can be achieved through the recruitment of various repair pathway complexes, to produce crossovers (CO) or noncrossovers (NCO). The fact that CO formation occurs with tight precision, coupled with the observation that only a small subset of total DSBs will become COs, suggests that orchestration of DSB repair events, and the various repair pathways that give rise to either CO or NCO events, is highly regulated at the molecular level (reviewed by [17]).

Two pathways have been defined for CO formation, the first involving the so-called Class I or “*ZMM*” pathway (for ZIP3, MSH4/5 and MER3), and the second, Class II pathway, involving the *Mus81* endonuclease, which functions as a heterodimer with EME1 (Mms4 in yeast) [18]–[21]. In *S. cerevisiae*, and possibly in the mouse, this Class II pathway appears to be restricted to a subset of DSBs that may be aberrant in structure and/or that may be processed initially by the RecQ helicase, Sgs1/BLM (yeast/mouse ortholog; [20]–[23]). These aberrant DSB repair intermediates (or joint molecules, JMs) include a variety of structures that result from secondary strand invasion events, usually involving independent activities of each end of the DSB, and/or from closely spaced DSBs. These aberrant JMs have been demonstrated biochemically in *S. cerevisiae* [20]–[23], but not yet in mammals. Under wildtype conditions in budding yeast, Sgs1 can disassemble and/or process many of these aberrant DSB repair intermediates towards NCO or Class I CO fates [20], [21]. However, a small proportion of them cannot be processed in this manner and thus become the target of MUS81-driven crossing over.

In the mouse, the class I pathway accounts for some 90–95% of COs, and the major intermediate marker for these events is the accumulation, in pachynema, of the MutL homolog heterodimer, MLH1 and MLH3 [24]–[27]. The remaining events are processed via the MUS81-dependent Class II pathway [27], [28]. Interestingly, however, our studies in mouse have demonstrated that the loss of *Mus81* results in the recruitment of 5–10% additional MLH1/MLH3 foci during pachynema, and that these additional foci may act to maintain CO rates at normal levels in the absence of MUS81 [28]. This suggests that a unique, possibly mammalian-specific, level of integration exists between the two crossover pathways. Importantly, BTBD12 interacts with many of the key players in both CO pathways, including BLM (reviewed by [29]), leading us to hypothesize that BTBD12 may functionally integrate the different CO pathways during mammalian meiosis.

We obtained a mouse line from the European Conditional Mouse Mutagenesis Program (EUCOMM), harboring a *Frt*-flanked βGeo cassette upstream of *LoxP*-flanked exon 3 of *Btbd12* gene, and as initially described by Crossan *et al* [30]. We call this conditional genetrap allele *Btbd12^βGeoFlox^*. In wildtype mice, BTBD12 protein localizes to spermatogonial and spermatocyte populations of the adult testis and is dramatically down-regulated at these sites in the mutant animals. Weak staining against BTBD12 persists, however, in the *Btbd12^βGeoFlox/βGeoFlox^* testes, but the protein fails to be recruited to meiotic chromosome cores to detectable levels. Despite residual protein, the mice are sub-fertile as a result of a reduced spermatogonial population coupled with failure to progress normally through meiotic prophase I resulting in less than 15% normal numbers of spermatozoa. Importantly, BTBD12 localization to meiotic chromosomes appears to be dependent on ATM, since the presence of BTBD12 protein is lost in *Atm^−/−^* males, and *Btbd12^βGeoFlox/βGeoFlox^* mice show genomic instability similar to that seen in the absence of ATM. Taken together, our data suggest that BTBD12 is a key substrate of ATM in the mammalian germ line, playing a role in the spermatogonial stages of gametogenesis, as well as in the entry into, and progression through, prophase I.

## Results

### BTBD12 protein localizes to spermatogonia and spermatocytes of the mouse testis

**Figure 1:**
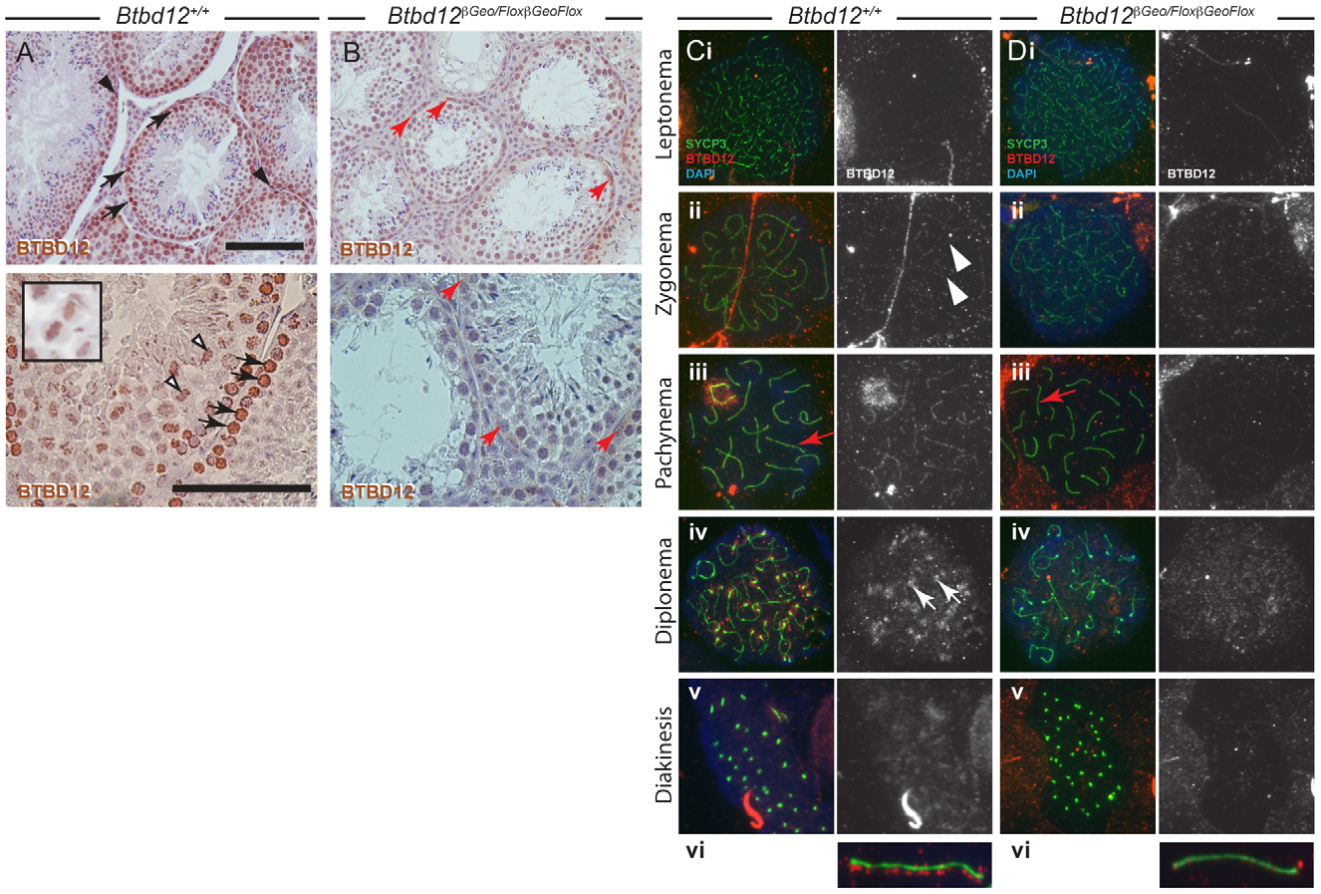
BTBD12 localization in wild type and *Btbd12^βGeoFlox/βGeoFlox^* spermatogenesis. (A, B) Testis sections from wild type (A) or *Btbd12^βGeoFlox/βGeoFlox^* mice (B) were stained with an antibody against BTBD12 (brown). BTBD12 is localized to spermatogonia (black arrowheads), prophase I spermatocytes (black arrows) and metaphase I spermatocytes (white arrowheads, and inset) in wild type sections, however only a small amount of residual staining is seen in the *Btbd12^βGeoFlox/βGeoFlox^* sections, in cells along the basal membrane of the tubule (red arrows). Scale bars are 100 µm. (C, D) BTBD12 localization in prophase I substages in wild type (Ci-vi) and *Btbd12^βGeoFlox/βGeoFlox^* (Di-vi) spermatocytes. Cells were stained with antibodies against SYCP3 (green) and BTBD12 (red), while DNA was stained with DAPI (blue). BTBD12 staining is shown alone in the grey panels. BTBD12 accumulates along chromosome cores during zygonema in wild type cells (Cii, white arrowheads), persists through pachynema (Ciii, vi) and remains only at the centromeres in diplonema (Civ, white arrows). Single pachytene chromosomes stained with SYCP3 (green) and BTBD12 (red) from wild type (Cvi) and *Btbd12^βGeoFlox/βGeoFlox^* (Dvi) mice are shown, with the red channel slightly offset from the green channel to better visualize the BTBD12 localization on the chromosome cores. The same chromosomes are shown on the original images (Ciii, Diii, red arrows).

Wildtype testis sections were stained with a commercial anti-BTBD12 antibody from Novus Biologicals that was raised against amino acids 1650–1700 of human BTBD12 at the c-terminus of the protein (Figure 1A, 1B). BTBD12 is expressed ubiquitously in the mouse, and is found in both adult testis and fetal ovary [8]. In adult testis of wildtype mice, the protein was found to localize strongly to spermatogonia and spermatocytes (Figure 1A, black arrowheads and arrows, respectively). At earlier stages of spermatogenesis, prior to entry into meiosis, the signal for BTBD12 protein appears to localize through the nucleus, with increased intensity of signal around the nuclear periphery. Upon entry into prophase I, the BTBD12 signal becomes more punctate in nature, associating with the increasingly condensed chromatin, but continuing to occupy the majority of the nuclear space. Additionally, BTBD12 protein localized to bivalent chromosomes on the meiotic spindle during metaphase of meiosis I (Figure 1A, white arrowheads and inset panel).

BTBD12 localization was evaluated on chromosome spread preparations of prophase I spermatocytes. In all chromosome “spread” preparations presented herein, we utilize the major protein component of the axial element of the synaptonemal complex (SC, the meiosis specific structure that assembles along and between each homologous chromosome pair), SYCP3, to visualize meiotic chromosome cores. We utilized an affinity-purified antibody that targets amino acids 1–350 of murine BTBD12 (antibody “NT”). The chromosome spreads from wildtype adult males (Figure 1 Ci-vi) showed accumulation of BTBD12 protein in a punctate staining pattern along the chromosome cores in zygonema (Figure 1 Cii), becoming more intense by pachynema (Figure 1Ciii). In some cases, increased staining was observed within the sex body (94% of wildtype cells having BTBD12 in this domain, compared to 0% in the *Btbd12* mutants; n of 33 and 31, respectively), in line with the fact that BTBD12 is a target of ATM, which plays a significant role in the formation of this sub-nuclear domain during mid-prophase I [31], [32]. One autosomal chromosome from a pachytene cell of each genotype is shown, enlarged, with the BTBD12 staining offset from the SYCP3 staining to facilitate visualization of the punctate pattern of the Btbd12 signal (Figure 1Cvi). By diplonema, BTBD12 was no longer present on the cores, but remained associated with the centromeres (Figure 1Civ), and had disappeared from the nucleus by diakinesis (Figure 1Cv).

### *Btbd12^βGeoFlox/βGeoFlox^* mutant testes show some residual BTBD12 protein and an absence of detectable BTBD12 on meiotic chromosome cores

The *Btbd12^βGeoFlox^* allele contains an *Frt*-flanked βGeo insertion into the *Btbd12* locus upstream of *LoxP*-flanked exon 3. The expectation, therefore, is that this allele would act as a gene trap, reducing expression of wildtype *Btbd12* mRNA. Consistent with this, in the *Btbd12^βGeoFlox/βGeoFlox^* mutant testis sections, we observe weak BTBD12 protein staining persisting in some cells, particularly those close to the basal membrane of the tubules, which are presumptive spermatogonia (Figure 1B, red arrows). This persistent staining, however, was much fainter than that seen in the wildtype testis sections, indicating a significantly lower abundance of BTBD12 protein in the mutant animals, and suggesting a splicing event around the βGeo cassette to produce BTBD12 protein. Importantly, there was no staining evident on metaphase chromosomes in the *Btbd12^βGeoFlox/βGeoFlox^* testes.

In spermatocytes from *Btbd12^βGeoFlox/βGeoFlox^* mutant littermates, BTBD12 did not appear to localize to chromosome cores with as intense a signal as in wildtype cells (Figure 1Di-vi), although faint staining was observed at pachynema in the *Btbd12^βGeoFlox/βGeoFlox^* mutants. This staining, however, was barely detectable above background, even at higher magnifications (Figure 1Diii and vi). Thus, the persistent BTBD12 signal observed in testis sections from *Btbd12^βGeoFlox/βGeoFlox^* males is not associated with prophase I chromosome cores, or is present at levels that are undetectable on chromosome spreads.

### Altered BTBD12 protein expression/stability in *Btbd12^βGeoFlox/βGeoFlox^* testis

**Figure 2:**
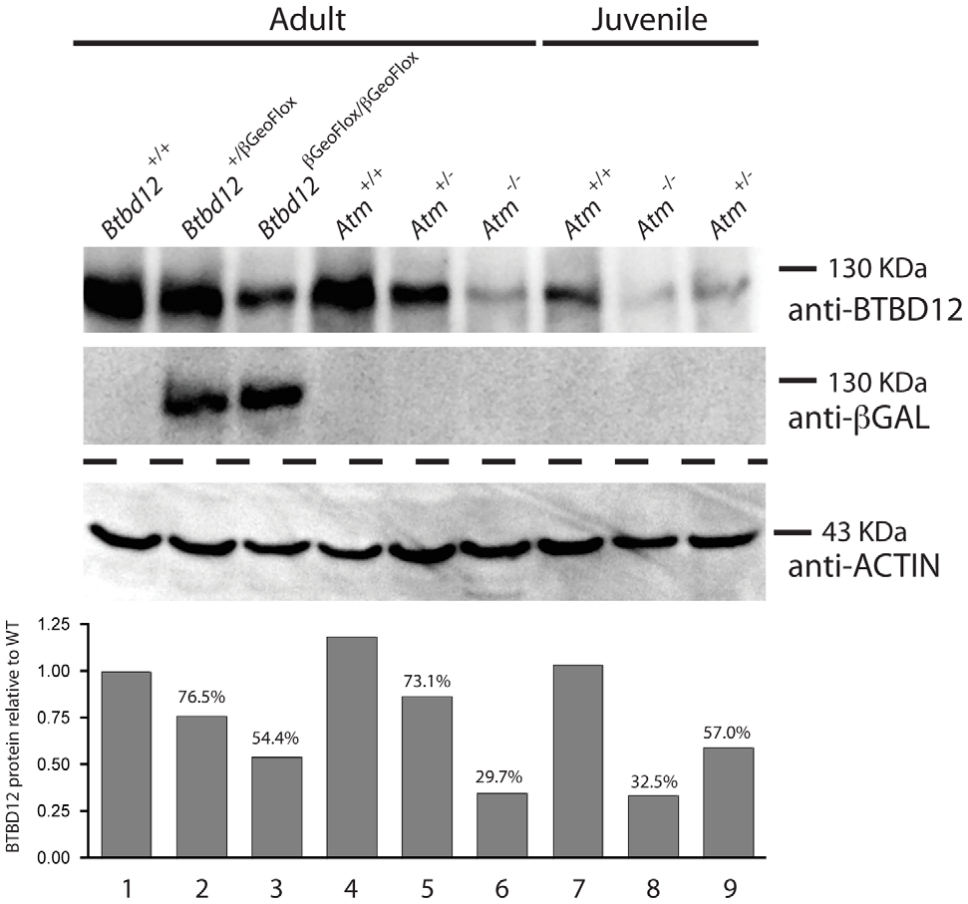
BTBD12 protein is down regulated in the *Btbd12^βGeoFlox/βGeoFlox^* mutant and the *Atm* null mice. Western blot analysis of whole testis protein from adult *Btbd12^+/+^*, *Btbd12^+/βGeoFlox^*, *Btbd12^βGeoFlox/βGeoFlox^* mice (lanes 1–3), *Atm^+/+^*, *Atm^+/−^*, *Atm^−/−^* adult mice (lanes 4–6) and *Atm^+/+^*, *Atm^−/−^*, *Atm^+/−^* juvenile mice (lanes 7–9) using antibodies against BTBD12, β-Galactosidase and Actin (protein control). Protein levels for BTBD12 were normalized against Actin, and are shown as a percentage of the BTBD12 found in each wild type control.

To confirm the presence of BTBD12 protein in wild type testes, and to explore the status of BTBD12 protein in the homozygous mutant animals, western blots were performed using whole testis protein extracts from adult and juvenile animals (Figure 2). Two antibodies were utilized, one C-terminal and one N-(not shown) terminal, as described above. Both antibodies produced a band of similar size in protein extracts from wild type adult testis (lane 1), with decreasing amounts observed in *Btbd12^βGeoFlox/+^* heterozygotes (lane 2) and further reduced expression observed in *Btbd12^βGeoFlox/βGeoFlox^* mutant protein extracts (lane 3). Quantitation of BTBD12 protein levels in testis extracts from all three genotypes revealed a decrease from wildtype levels of 23.5% and 45.6% for heterozygotes and homozygous mutant samples, respectively (Figure 2 graph). The presence of residual 130 kDa protein in the homozygous mutant animals is in line with the persistent protein signal observed in immunohistochemical staining of testis sections from these animals (Figure 1B), and suggests that the mutant allele is transcribed at a reduced level compared to the wildtype allele. As expected, the lanes containing *Btbd12^βGeoFlox/+^* and *Btbd12^βGeoFlox/βGeoFlox^* protein (lanes 2 and 3 in Figure 2) also show positive staining for β-galactosidase at approximately the same size. No β-galactosidase band is observed in the wildtype testis protein lane. Taken together, these results show that the BTBD12 protein present in testis extracts from *Btbd12^βGeoFlox/+^* and *Btbd12^βGeoFlox/βGeoFlox^* males encompasses some portions of both the N-terminus and C-terminus of the protein, and most likely arises as a result of splicing around the FRT-flanked cassette, but that expression of this protein is dramatically reduced by the presence of the βGeo cassette. Moreover, βGeo cassette itself is also transcribed from the targeted allele, either as a distinct protein and/or as a fusion with BTBD12. We favor the former option given that the size of the BTBD12 and βGeo bands are similar.

### *Btbd12^βGeoFlox/βGeoFlox^* mice are viable, comparable in body weight to wildtype littermates, and are prone to anophthalmia and microphthalmia

*Btbd12^βGeoFlox/βGeoFlox^* mutant mice are viable and are born at slightly lower than expected Mendelian rates. Out of a total of 395 pups born in 65 litters from heterozygote breedings, 66 of them were mutants, compared with an expected frequency of 98.75 (Table S1). These data are significantly different, at the p<0.05 level, from the expected percentages mutant or wildtype numbers (P = 0.012, χ^2^ test). This rate is slightly higher than that seen in the previous report describing these mice [30]. Occasionally we observe that mutants exhibit lower birth weights than their wildtype litter mates (not shown) but, by adulthood, these animals have regained weights comparable to their siblings. Thus, *Btbd12^βGeoFlox/βGeoFlox^* adult mice were not significantly smaller than wildtype littermates (average weights 19.5 and 21.3 g, respectively P = 0.25, unpaired T-test) but exhibited varying degrees of anophthalmia and microphthalmia from birth. 33 out of 66 (50%) mutants showed defects of one or both eyes. No such deformities were apparent in wildtype litter mates indicating a possible role for BTBD12 in eye development and in line with previous reports describing this EUCOMM mouse line [30].

### *Btbd12^βGeoFlox/βGeoFlox^* mutants have a higher level of genomic instability than their wildtype littermates

It is well known that mammalian species with mutations in *Atm* show an increase in genomic instability (GIN), including defects in cell cycle regulation, sensitivity to DNA damage-inducing agents, and chromosomal aberrations (reviewed by [33]). Since BTBD12 is believed to be a direct target for phosphorylation by ATM/ATR, we assessed both wild type and *Btbd12^βGeoFlox/βGeoFlox^* mutant mice for GIN. A micronucleus formation assay was performed on peripheral blood from both *Btbd12^βGeoFlox/βGeoFlox^* mutants and wild type littermate controls [34], [35]. The *Btbd12^βGeoFlox/βGeoFlox^* mutant animals showed over a two-fold increase in micronucleus formation compared with wild type mice, which was statistically significant (Figure S1; p<0.0001). This result indicates that these mutants have a high level of GIN in their somatic cells approaching, though not as great, as that seen in *Atm* null mice [36].

### Male *Btbd12^βGeoFlox/βGeoFlox^* mice show reduced fertility associated with a paucity of spermatogenic cells in the testis

**Figure 3:**
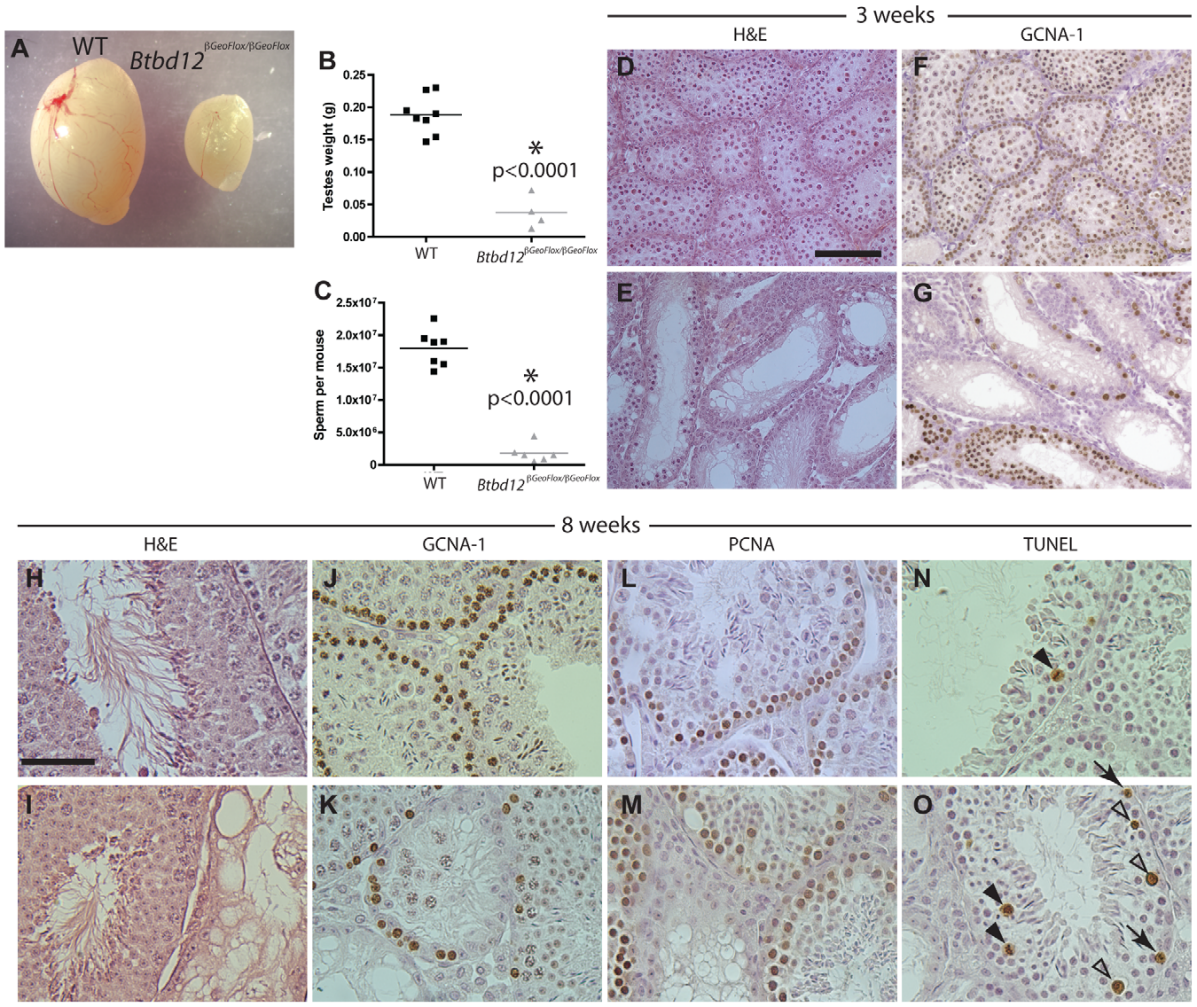
*Btbd12^βGeoFlox/βGeoFlox^* mutants have reduced testicular germ cell proliferation. (A) Testes from *Btbd12^βGeoFlox/βGeoFlox^* mice are severely reduced in size compared with wild type littermates. (B) Testes weights of wild type (black) and *Btbd12^βGeoFlox/βGeoFlox^* mutant (grey) mice (P<0.0001). (C) Eipdidymal sperm numbers in wild type (black) and *Btbd12^βGeoFlox/βGeoFlox^* mutant (grey) mice (<0.0001). (D–G) Testis sections from wild type (D, F) or *Btbd12^βGeoFlox/βGeoFlox^* mutant (E, G) 3 week old mice, stained with either H&E (D–E) or an antibody against GCNA-1 (F–G, brown). (H–O) Testis sections from wild type (H, J, L, N) or *Btbd12^βGeoFlox/βGeoFlox^* mutant (I, K, M, O) 8 week old mice, stained with H&E (H–I), anti-GCNA-1 (J–K), anti-PCNA1 (L–M) or TUNEL (N–O). TUNEL stained apoptotic cells (N, O, arrowheads) were staged as either spermatocytes prior to metaphase I (white arrowheads) or at metaphase I (black arrowheads). D–G Scale bar is 50 µm. H–O scale bar is 100 µm.

*Btbd12^βGeoFlox/βGeoFlox^* mutant mice showed significantly decreased testis size when compared to wildtype littermates (approximately 25%, Figure 3A, 3B, p<0.0001). Sperm counts performed on the two cohorts of mice revealed that *Btbd12^βGeoFlox/βGeoFlox^* mice have only about 10% of the amount of epididymal sperm found in wildtype littermates (Figure 3C), and exhibit dramatically reduced fertility, with only 3 litters born to two different *Btbd12^βGeoFlox/βGeoFlox^* males over the period of 9 months, with the youngest male to sire a litter being approximately 7 weeks old. Female mutants were sterile, with two mutant females bred to a fertile male, over a period of a year, yielding no pregnancies, while wildtype cage mates produced healthy offspring from the same male.

H&E staining of testis sections from both wildtype and *Btbd12^βGeoFlox/βGeoFlox^* mutant mice at both 3 and 8 weeks of age (Figure 3D, 3E, 3H, 3I) showed that the seminiferous tubules of *Btbd12^βGeoFlox/βGeoFlox^* males are extremely variable in their cell density and also in the progression through meiosis. For example, in 8-week old *Btbd12^βGeoFlox/βGeoFlox^* mutant mice, neighboring tubules showed almost normal tubule morphology, juxtaposed to almost empty, abnormal tubules (Figure 3I). Immunohistochemical staining with the spermatogonial and early spermatocyte cell marker, GCNA-1, showed a severe depletion of early germ cells within the tubules of the *Btbd12^βGeoFlox/βGeoFlox^* compared with those within the wildtype litter mate mice, at both 3 week and 8 weeks of age (Figure 3F, 3G, 3J, 3K). By contrast, the proliferating cell marker, PCNA, showed a similar staining pattern in the majority of tubules in both wildtype and *Btbd12^βGeoFlox/βGeoFlox^* mice (Figure 3L, 3M) suggestive of normal progression through spermatogonial divisions and in self-renewal capabilities. The maintainenance of PCNA signal in spite of reduced PGC pool is suggestive of prolonged S-phase. In line with this, we observed increased TUNEL labeling of apoptotic cells in testis sections of *Btbd12^βGeoFlox/βGeoFlox^*, mostly during meiosis I. The majority of these cells are undergoing apoptosis at around the time of exit from prophase I, but some also appear to be in mid-prophase (Figure 3N, 3O, arrowheads). In a few instances, some TUNEL-positive cells appear to be in pre-meiotic stages (Figure 3O, arrows), but these are clearly fewer in number than those apoptotic cells in prophase I. Collectively, these results demonstrate a loss of germ cells from the testis of *Btbd12^βGeoFlox/βGeoFlox^* males, starting as early as the first wave of meiotic entry within the first three weeks of postnatal life.

### Male *Btbd12^βGeoFlox/βGeoFlox^* mice show declining primordial germ cell numbers within their testes from embryonic day 18 onwards

**Figure 4:**
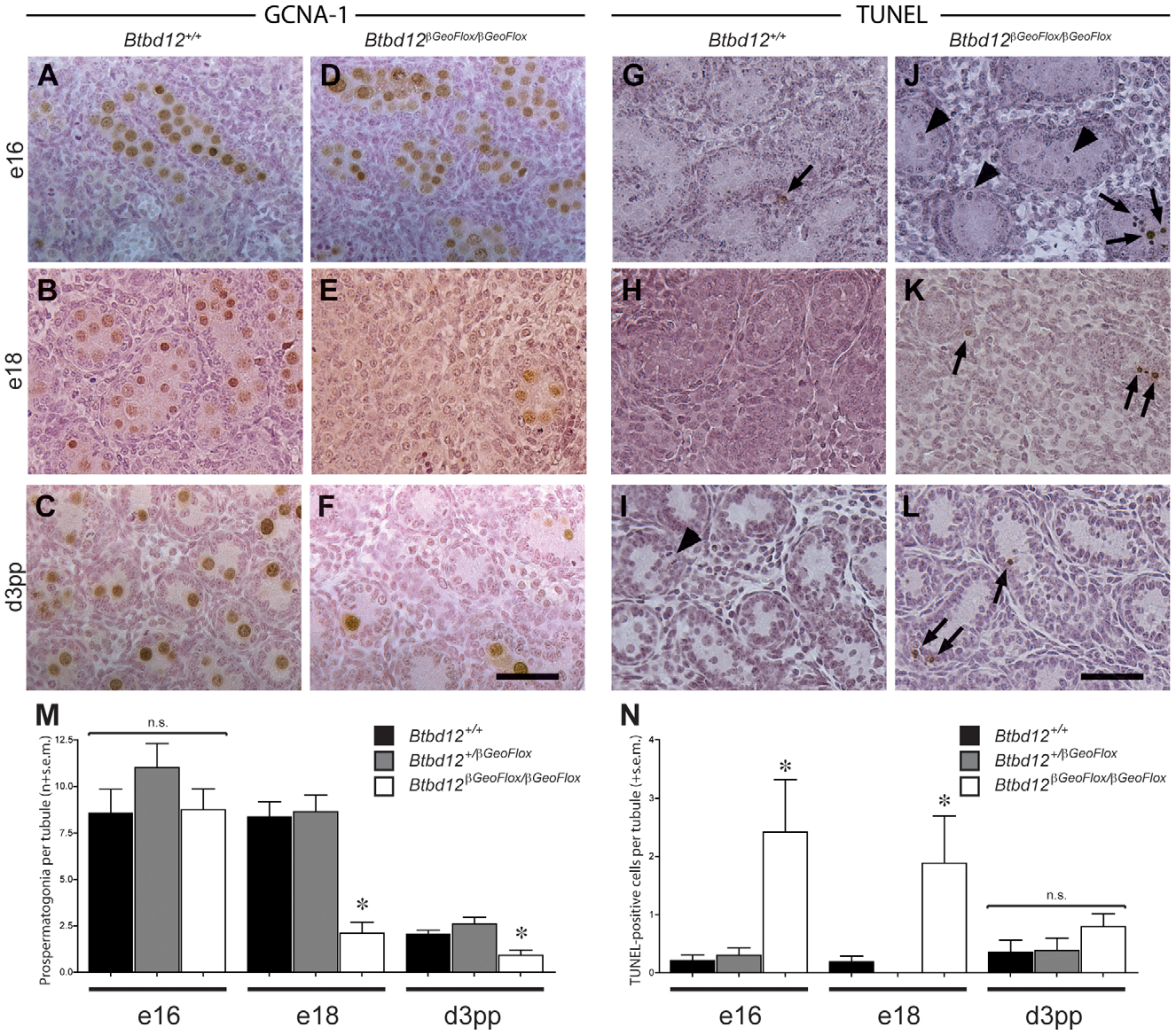
Spermatogonial germ cell proliferation in prepubescent males. Testes sections from either wildtype (A–C, G–I) or *Btbd12^βGeoFlox/βGeoFlox^* (D–F, J–L) mice from three different ages (day e16, day e18, day 3 pp) were stained with either GCNA-1 (A–F) or TUNEL labeled (G–L). TUNEL labeled apoptotic spermatogonia are shown by the arrows. Arrowheads mark mitotic TUNEL-positive cells. Scale bar is 50 µm. Quantitation of the GCNA-1 labeled (M) and TUNEL-positive (N) cells reveals a statistically significant decrease in prospermatogonia, accompanied by an increase in apoptotic cells (asterisks).

Given the apparently normal proliferative capacity of spermatogonia in testes of 8-week old *Btbd12^βGeoFlox/βGeoFlox^* mice, we questioned why the tubules of 3 week-old mice were so heterogeneous with respect to cellular density. If a failure of spermatogonial proliferation is not the cause of the lack of cellularity of certain tubules in the *Btbd12^βGeoFlox/βGeoFlox^* males, then a second possibility is that the testis of these mice fail to be populated with appropriate numbers of spermatogonial precursors, known as pro-spermatogonia or gonocytes, during development. To investigate this option, testes were obtained for both wildtype and *Btbd12^βGeoFlox/βGeoFlox^* males between embryonic (e) day 18 and day 3 post-partum (pp), and prospermatogonia were visualized with antiserum against GCNA-1. In wildtype males, the G_0_-arrested prospermatogonia population is established around embryonic day 12.5–16.5 following migration of the primordial germ cells (PGC) to the genital ridge [37], [38], the exact timing being somewhat controversial, and remain quiescent until just prior to birth [39]. During the period between e18 and day 3pp, a large number of prospermatogonia are lost by apoptosis. During that time, approximately 1 to 7 prospermatogonia may be observed within the seminiferous cords of the developing testis, and these cells then start to proliferate from day 4 pp onwards [39]. In *Btbd12^+/+^* males, these large round cells appeared separated from the basement membrane by the Sertoli cells, which are more columnated in appearance, and they stained readily with numerous markers including GCNA-1 (Figure 4A–4C, brown cells) and mouse Vasa homolog (MVH; not shown) from e16 onwards. By day 3 pp, every testis cord section contains on average 2.08±0.19 prospermatogonia per cord, representing a range of 1 to 10 cells (Figure 4C, 4M; Table S2), having declined dramatically at around the time of birth. In *Btbd12^βGeoFlox/βGeoFlox^* males at e16, normal numbers of GCNA-1 positive prospermatogonia are observed (Figure 4D), but by e18, their numbers have declined significantly (Figure 4E, 4M; Table S2). By d3 pp, the majority of testis cords contain no prospermatogonia, with a mean prospermatogonia content of 0.92±0.28 (Figure 4F, 4M; Table S2). The Sertoli cell populations in both *Btbd12^+/+^* and *Btbd12^βGeoFlox/βGeoFlox^* males appeared normal throughout (Figure 4B, 4D, 4F). In line with this reduced cellularity within the testicular cords, we observed a marked increase in apoptosis, as measured by TUNEL labeling of testis sections from wildtype (Figure 4G–4I, 4N) and mutant (Figure 4J–4L, 4N, arrows) animals, particularly at e16 and e18 (Figure 4J, 4K, 4N; Table S2). These data demonstrate that the population of spermatogonia within the testes of *Btbd12^βGeoFlox/βGeoFlox^* males is markedly lower than that seen in wildtype as a result of early loss of these cells after arriving at the genital ridge, and suggesting that the proliferation of PGCs in the developing testis is dependent on BTBD12.

### Male *Btbd12^βGeoFlox/βGeoFlox^* exhibit altered progression through prophase I

**Figure 5:**
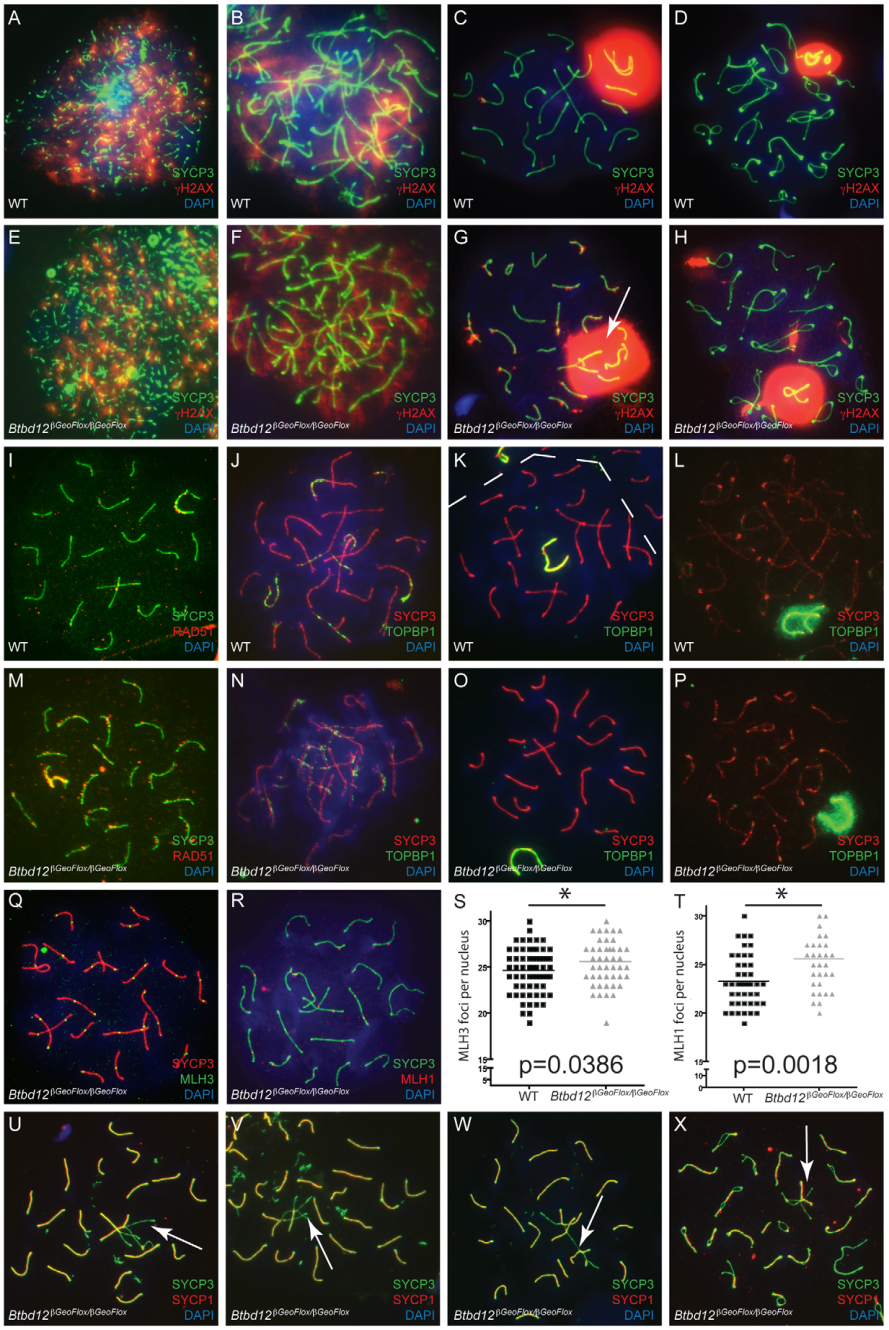
*Btbd12^βGeoFlox/βGeoFlox^* spermatocytes show altered meiotic progression. (A–H) Meiotic chromosome spreads from wild type (A–D) and *Btbd12^βGeoFlox/βGeoFlox^* mutants (E–H) were stained with antibodies against SYCP3 (green) and γH2AX (red), and DNA was stained with DAPI (blue). Abnormal sex body morphology in pachynema in the *Btbd12^βGeoFlox/βGeoFlox^* mutant is shown by the white arrow. (I, M) Meiotic chromosome spreads from wild type (I) and *Btbd12^βGeoFlox/βGeoFlox^* mutants (M) were stained with antibodies against SYCP3 (green) and RAD51 (red), and DNA was stained with DAPI (blue). Meiotic chromosome spreads from wild type (J–L) and *Btbd12^βGeoFlox/βGeoFlox^* mutants (N–P) were stained with antibodies against SYCP3 (red) and TOPBP1 (green), and DNA was stained with DAPI (blue). The dashed line represents the boundary between two cells (K). (Q) Meiotic chromosome spreads *Btbd12^βGeoFlox/βGeoFlox^* mutants were stained with antibodies against SYCP3 (red) and MLH3 (green) and DAPI (blue). (R) Meiotic chromosome spreads *Btbd12^βGeoFlox/βGeoFlox^* mutants were stained with antibodies against SYCP3 (green) and MLH1 (red) and DAPI (blue). (S) MLH3 focus counts per cell nucleus in wild type (black) and *Btbd12^βGeoFlox/βGeoFlox^* mutants (grey). (T) MLH1 focus counts per cell nucleus in wild type (black) and *Btbd12^βGeoFlox/βGeoFlox^* mutants (grey). (U–X) Meiotic chromosome spreads *Btbd12^βGeoFlox/βGeoFlox^* mutants were stained with antibodies against SYCP3 (green) and SYCP1 (red) and DAPI (blue). Regions of asynapsis are shown by the white arrows.

To examine meiotic prophase I progression, chromosome spreads from both *Btbd12^βGeoFlox/βGeoFlox^* and wildtype spermatocytes were stained with antibodies against SYCP3 and γH2AX (Figure 5A–5H), as a marker for DNA DSBs during prophase I. γH2AX accumulated on leptotene spermatocytes similarly in both wildtype and *Btbd12^βGeoFlox/βGeoFlox^* cells (Figure 5A, 5E), demonstrating the appearance and processing of DSB events. By zygonema, however, γH2AX localization began to diminish on the chromosome cores of wildtype spermatocytes (Figure 5B), coincident with the onset of DSB repair processes. By pachynema, and into diplonema, γH2AX localization was only restricted to a strongly-stained domain coincident with the sex body (Figure 5C, 5D and [40], [41]). By contrast, γH2AX staining is apparent at zygonema in the *Btbd12^βGeoFlox/βGeoFlox^* spermatocytes (Figure 5F), but persists along the autosomes well into pachynema, at which time this staining is limited to the sex body in wildtype cells (Figure 5C, 5G; ). γH2AX staining remains apparent well into diplonema in the *Btbd12* mutants (Figure 5D, 5H; Figure S2).

To investigate the progression of DSB repair, we assessed RAD51 distribution along SCs during prophase I. Specifically, we were interested in observing persistence of RAD51 signal in *Btbd12^βGeoFlox/βGeoFlox^* spermatocytes as an indication of unrepaired, or delay in repair of, DSBs. As expected, we observed progressive loss of RAD51 from SCs in wildtype spermatocytes entering pachynema (Figure 5I; Table S3), whereas RAD51 focus numbers remained elevated through pachynema in *Btbd12^βGeoFlox/βGeoFlox^* spermatocytes (Figure 5M; Table S3). This difference between wildtype and mutant spermatocytes in terms of pachytene RAD51 foci (means of 26.2 and 45.1, respectively) was statistically significant.

The BRCT domain-containing protein, TOPBP1, functions in replication and DNA damage checkpoint processes, and in meiosis, it localizes to sites of DNA damage in response to DSBs [42], [43]. TOPBP1 is also known to be required for ATR binding/activation in a number of organisms [44]–[46] and, along with ATM/ATR kinases, may be a part of the machinery that monitors recombination during prophase I and activates the meiotic checkpoint. Indeed, TOPBP1 localizes exclusively to sites of SPO11-induced DSB, as demonstrated by co-localization with γH2AX [47].

Despite the massive increase in γH2AX staining, *Btbd12^βGeoFlox/βGeoFlox^* spermatocytes showed no difference in the TOPBP1 localization pattern compared to that seen in chromosome spreads from wild type spermatocytes (Figure 5J–5P). TOPBP1 accumulated on synapsed chromosomes during zygonema, and gradually decreased until it remained only at the sex chromosomes during pachynema, indicating that this signaling pathway is not affected by the loss of BTBD12 from the chromosome cores, in contrast to the persistent RAD51 observed on SCs from *Btbd12^βGeoFlox/βGeoFlox^* spermatocytes.

MLH1 and MLH3 localization was used to examine the progression of DSB repair events via the “ZMM”, Class I CO pathway (Figure 5Q–5T), which is overseen by key members of the DNA mismatch repair (MMR) family: MSH4, MSH5, MLH1 and MLH3. MLH1 and MLH3 form a heterodimer that binds to the MSH4/MSH5 heterodimer in pachynema [48]–[52]. MSH4/MSH5 assemble on DSB repair sites in zygonema in numbers that, in mice at least, far exceed the final tally of chiasmata [53], [54]. The number of these foci is then pared down through prophase I, but still maintains levels that are approximately two-fold higher than the final chiasmata count [53], [54]. Association of MLH1/MLH3 with a subset of these sites is thought to stabilize these events, resulting in the resolution of these structures via the class I CO pathway [24]–[26].

In spermatocyte spreads from both wildtype and *Btbd12^βGeoFlox/βGeoFlox^* males, MLH1 and MLH3 foci arise at pachynema, at frequencies of 1–2 foci per chromosome, which is comparable to that seen previously in wildtype [25], [26]. The temporal and spatial dynamics of MLH1 and MLH3 association with the SYCP3-positive chromosome core was similar for wild type and *Btbd12^βGeoFlox/βGeoFlox^* spermatocytes (Figure 5Q–5T), suggesting similar progression of class I CO events. When foci were counted and compared between wildtype and mutants, however, we observed a slight, yet statistically significant, increase in foci number for both MLH1 and MLH3 in *Btbd12^βGeoFlox/βGeoFlox^* spermatocytes, equating to approximately 1 additional focus per nucleus for each (Figure 5S, 5T, p = 0.0018 for MLH1 and p = 0.0389 for MLH3, unpaired T test). For MLH1, the mean number of foci was 23.28 and 25.56 for wildtype and *Btbd12^βGeoFlox/βGeoFlox^* males, respectively (Table S3). For MLH3, the mean number of foci was 24.62 and 25.57 for wildtype and *Btbd12^βGeoFlox/βGeoFlox^* males, respectively. MLH1 foci were also significantly elevated in meiotic spreads from female day e19 embryos, with average MLH1 focus numbers of 22.29 and 24.00 in wildtype and *Btbd12^βGeoFlox/βGeoFlox^*, respectively (p = 0.02, data not shown). The earlier recombination intermediate MSH4 remained the same in both wildtype and *Btbd12^βGeoFlox/βGeoFlox^* spermatocytes (data not shown).

To assess synapsis, chromosome spreads from *Btbd12^βGeoFlox/βGeoFlox^* spermatocytes were stained with an antibody against the central element component, SYCP1 (Figure 5U–5X). Interestingly, at pachynema, approximately 10% of cells harboring the mutant *Btbd12* locus showed abnormal synapsis, compared to less than 1% for wildtype cells (not shown), characterized by frequent pairing between more than two chromosomes, incomplete synapsis at pachynema, and synapsis between chromosomes of differing lengths (Figure 5S–5U). In some cases, these synaptic errors appeared to persist into diplonema without first resulting in apoptosis (Figure 5X).

### *Btbd12^βGeoFlox/βGeoFlox^* chromosomes form chiasmata at normal rates and show no defects in chromosome segregation at meiosis I in males and females

**Figure 6:**
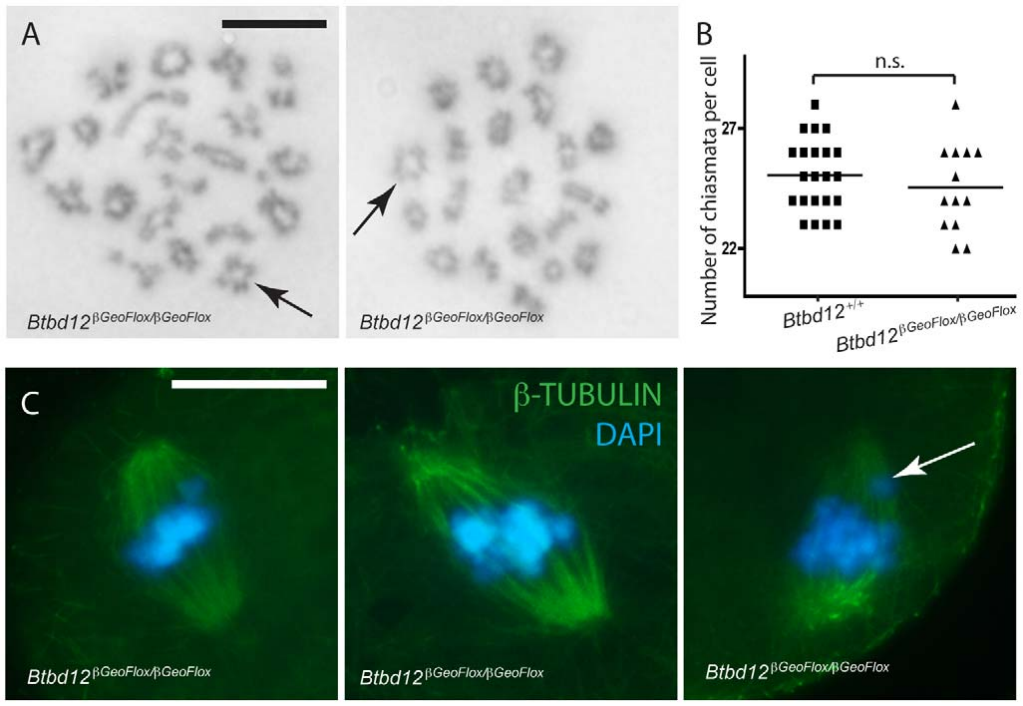
Crossovers form in *Btbd12^βGeoFlox/βGeoFlox^* mutants and there is no evidence of spindle defects. (A) Diakinesis preps from *Btbd12^βGeoFlox/βGeoFlox^* spermatocytes show evidence of chiasmata (black arrows). Scale bar is 10 µm. (B) Chiasmata counts from *Btbd12^+/+^* (black squares) and *Btbd12^βGeoFlox/βGeoFlox^* (black triangles) diakinesis preparations show no significant difference (p = 0.2) (C) *Btbd12^βGeoFlox/βGeoFlox^* oocytes at metaphase I show no irregularities in chromosome alignment on the metaphase spindle. Although misaligned chromosomes were observed on occasion (white arrow), these were seen in wild type oocytes at the same frequency. Scale bar is 20 µm.

To assess the impact of loss of BTBD12 on the first meiotic division, we prepared air-dried diakinesis chromosome spreads and stained them with Giemsa. Chiasmata formation occurred in both wild type (not shown) and *Btbd12^βGeoFlox/βGeoFlox^* spermatocytes (Figure 6A). The number of diakinesis stage cells was severely depleted in *Btbd12^βGeoFlox/βGeoFlox^* mice, suggesting loss of these cells prior to completing prophase I and/or delayed progression through to diplonema. Of the diakinesis cells that we obtained from *Btbd12^βGeoFlox/βGeoFlox^* males, however, none showed any changes in chiasmata counts compared to that seen in wildtype litter mates (Figure 6B).

To ask whether the number of chiasmata present in *Btbd12^βGeoFlox/βGeoFlox^* mutant animals is sufficient to cause appropriate separation of chromosomes during the first meiotic division, an examination of oocytes undergoing metaphase I to anaphase I progression was undertaken. Oocytes from wildtype and *Btbd12^βGeoFlox/βGeoFlox^* mice were stained with an antibody against β-tubulin to show the meiotic spindle and DAPI to stain the DNA (Figure 6C). The arrangement of chromosomes on the meiotic spindle in *Btbd12^βGeoFlox/βGeoFlox^* mutant oocytes was similar to wild type controls, with 53 cells examined from each genotype. In both cases, occasional cells appear to show one or two misaligned chromosomes, but this occurs at similar rates in oocytes from wild type and *Btbd12^βGeoFlox/βGeoFlox^* females (Figure 6C, arrow; 3/53 cells for both wildtype and mutant). Taken together, these results indicate that, despite elevated MLH1/MLH3 focus numbers, chiasmata counts are unaffected in *Btbd12^βGeoFlox/βGeoFlox^* mutant animals. Moreover, these chiasmata can, and do, result in normal metaphase I progression, resulting in appropriate chromosome segregation at the first meiotic division. However, the fact that very few diakinesis cells are obtained suggests either a delay in prophase I completion or loss of cells through prophase I prior to diakinesis.

### BTBD12 localization to meiotic chromosome cores is disrupted in Atm null testes

**Figure 7:**
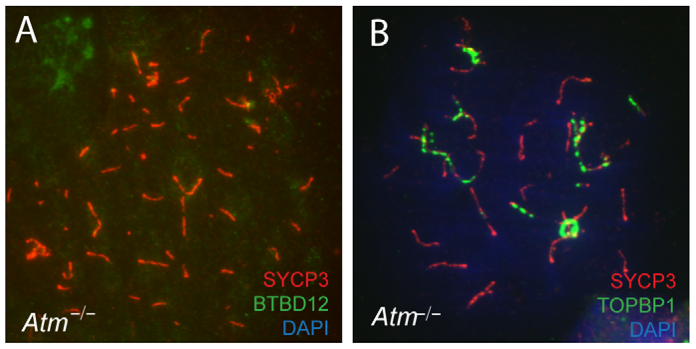
BTBD12 is down regulated in *Atm* null mice. (A) Spermatocyte from an *Atm^−/−^* null mouse stained with anti-SYCP3 (red), anti-BTBD12 (green) and DAPI (blue). (B) Spermatocyte from an *Atm^−/−^* null mouse stained with anti-SYCP3 (red), anti-TOPBP1 (green) and DAPI (blue).

BTBD12 was first identified as a potential kinase target of ATM [11]. To investigate the functional interaction between these two proteins, we examined the localization of BTBD12 on meiotic chromosomes in the absence of ATM. When co-immunostaining for BTBD12 and SYCP3 was performed on spread preparations from *Atm* null spermatocytes, we observed a complete absence of BTBD12 protein on chromosome cores (Figure 7A), compared to the punctate pattern of BTBD12 staining observed on chromosome cores wild type spermatocytes (Figure 1C). TOPBP1, however, localizes normally to the cores in *Atm* null cells (Figure 7B) in line with the demonstration in budding yeast that Mec1(ATR) activation is dependent on Dpb11(TOPBP1), rather than *vice versa*, and that Mec1, in turn, mediates only the functional interaction between Slx4(BTBD12) and Dpb11(TOPBP1), rather than regulating directly the localization of TOPBP1 [44], [55], [56].

BTBD12 protein is also down-regulated in *Atm* ^−/−^ males, when compared to the wildtype littermate controls, with *Atm^+/−^* males showing intermediate levels of BTBD12 protein (Figure 2, lanes 4–6). Testis protein extracts from *Atm* null mice show a decrease in BTBD12 protein to 70.3% of that seen in wildtype males, compared to a decrease to 26.9% in *Atm* heterozygotes (Figure 2 graph). Since ATM deletion results in pachytene meiotic failure in mice [57], [58], western blots were also performed on juvenile testis extracts at day 17 post-partum to ensure that all protein extracts from *Atm^+/+^*, *Atm^+/−^*, and *Atm^−/−^* contained equivalent cell populations. Indeed, even with higher proportions of leptotene and zygotene cells present in the day 17 extracts, protein from *Atm^−/−^* males showed a depletion of BTBD12 by 67.5% compared to *Atm^+/+^* males (Figure 2, lanes 7 and 8), with *Atm^+/−^*protein extracts showing an intermediate decrease of 43% compared to wildtype levels (lane 9). Thus, even when taking into account the earlier meiotic failure of *Atm^−/−^* males, BTBD12 protein is severely reduced in the absence of ATM.

## Discussion

The results presented herein describe, for the first time, the role of BTBD12 (SLX4) in mammalian gametogenesis. Our data show that BTBD12 plays dual roles in gametogenesis, firstly in facilitating primordial germ cell proliferation and establishment of the spermatogonial pool, possibly by ensuring genome stability, and secondly, in meiotic recombination events. These studies demonstrate that BTBD12 protein localizes to spermatogonia and spermatocytes of the testis. In the latter, BTBD12 is found along chromosome cores during prophase I, accumulating as early as zygonema and persisting through until late pachynema.

To explore the role of BTBD12 in mammalian gametogenesis, we obtained the *Btbd12^βGeoFlox/βGeoFlox^* mutant mouse line from the European Conditional Mouse Mutagenesis program (EUCOMM). The genetic disruption at the *Btbd12* allele results in residual protein that appears on western blots and, to a lesser extent, on immunohistochemical sections. The detected protein could reflect either a truncated fusion protein consisting of BTBD12 and βGeo, or intact BTBD12 protein generated by a splicing event that removes the βGeo cassette, retaining separate and identiable β-galactosidase protein expression. We favor this latter possibility because the BTBD12 protein detected in the mutant animals can be detected with antibodies against either the N- or the C-terminus. Further, cDNA analysis reveals the presence of every exon of the mouse gene in reverse transcribed RNA from *Btbd12^βGeoFlox/βGeoFlox^* testis (data not shown). The reduced intensity of BTBD12 signal in the *Btbd12^βGeoFlox/βGeoFlox^* testis extracts compared to wildtype extracts indicates that the presence of the βGeo cassette dramatically reduces the efficiency of BTBD12 protein production and/or reduces the stability of the protein. Importantly, the residual BTBD12 protein does not localize appropriately to meiotic chromosome cores, or localizes at levels that are undetectable using standard chromosome spreads, leading us to conclude that the functional activity of BTBD12 is abnormal in these mice, at least in the context of prophase I.

Micronucleus formation in *Btbd12^βGeoFlox/βGeoFlox^* mice is very much elevated compared to wildtype litter mates, in line with a recent report that first described this mouse line [30]. Crossan *et al* report that the phenotype of *Btbd12^βGeoFlox/βGeoFlox^* mice bears some resemblance to human Fanconi anemia (FA), including blood cell cytopenia and numerous developmental deformaties such as the anophthalmia reported herein. This report is in line with another recent publication demonstrating that human *SLX4* mutations are also found in a subset of FA patients [12]. Crossan *et al* also describe gonadal defects and subfertility in *Btbd12^βGeoFlox/βGeoFlox^* mice [30], but did not explore the origins of these phenotypes. They report that the histological appearance of the testes is consistent with a defect in meiosis, but they did not document pre-meiotic defects in these animals. They did, however, allude to similarities between the testicular phenotype of *Btbd12^βGeoFlox/βGeoFlox^* males and that of other FA-associated DNA repair proteins with which BTBD12 interacts, including *Ercc1*, *Fancd2*, *Fancl*, and *Fanca* [59]–. These latter mutations result in spermatogenic phenotypes ranging from spermatogonial proliferation defects, to meiotic defects, to defects in spermatozoa morphology. As will be discussed, results presented herein suggest that the phenotypic defects observed in the testes of *Btbd12^βGeoFlox/βGeoFlox^* males may be quite distinct from these other mutations.

Given the dramatic loss of testis weight we observe in the adult *Btbd12^βGeoFlox/βGeoFlox^* males, coupled with the severe paucity of cells in many seminiferous tubules as early as the period of the first wave of meiosis (days 13–26 pp), we reasoned that a large proportion of germ cells must be lost prior to entry into meiotic prophase I. Thus, these mice suffer from multiple germ cell defects, one involving spermatogonial proliferation and the other involving meiotic progression. The combined effect is a drop in testis size of 75% and a depletion of epididymal spermatozoa by about 90% of wildtype numbers. Given that we have observed only 3 viable pregnancies from females mated to *Btbd12^βGeoFlox/βGeoFlox^* males over a 9-month period, we conclude that these defects result in sub-fertility. Breeding data from *Btbd12^βGeoFlox/βGeoFlox^* females shows a more severe phenotype, with no pregnant females after 1 year of breeding. These data are more severe than the original description of these mice by the Crossan *et al* [30], but it should be noted that their analysis included many more mice over a much longer time period.

As predicted, histological examination of testis sections from e16 onwards revealed a dramatic decrease in the numbers of spermatogonial precursors from late gestation, known as prospermatogonia or gonocytes, within the developing seminiferous cords of *Btbd12^βGeoFlox/βGeoFlox^* male pups compared to their wildtype littermates. These data suggest that BTBD12 may function to promote DNA repair mechanisms during early proliferation of the PGC population within the developing gonad. PGCs originate at the posterior end of the primitive streak at e7, numbering around 45 [62]. They then migrate to the genital ridge, during which time they undergo a rapid cell proliferation to achieve a cell number of 3000 by e11.5 [63]. They continue to proliferate within the developing gonad until e13 when the testis/seminiferous cord structures form, trapping the now-mitotically arrested prospermatogonia within them [63]. They remain arrested until just prior to birth when they resume proliferation to provide the full complement of prospermatogonia to the post-natal testis. This late gestational wave of proliferation is also associated with increased germ cell apoptosis, even in testes from wildtype males. While the molecular pathways responsible for maintenance of genome integrity during this period are largely un-documented, it is likely that surveillance mechanisms exist similar to those found in somatic cells. Indeed, analysis of *Atm* mutant males reveals a requirement for ATM function in pre-meiotic spermatogonia [64]. Given the severe consequences of genomic instability within the PGC population for propagation of genetic mutations to offspring, it is plausible that particularly stringent DNA repair mechanisms must exist in these cells. Our results demonstrate that the PGCs arriving in the embryonic gonad appear normal in distribution and number since the testis of e16 mutants is similar in cellularity to that of wildtype littermates. From this time onwards, however, the numbers of prospermatogonia begin to decline rapidly in the testes of *Btbd12^βGeoFlox/βGeoFlox^* males such that the final number of these germ cells is dramatically lower in mutant males at e18 and at day 3 pp compared to wildtype males. This wave of apoptosis appears to coincide with the wave of proliferation that occurs around the time of birth because a large number of mitotic figures are observed in the testes of both wildtype and mutant males at this time (Figure 4, arrowheads).

Crossan *et al* have suggested that the germ cell defects present in *Btbd12^βGeoFlox/βGeoFlox^* mice may be similar to that of mutant mice for other key DNA repair genes, particularly those of various FA complementation groups [30]. Indeed, there are substantial similarities between these phenotypes described herein and those for proteins that have been shown to interact with BTBD12. For example, a targeted nullizygous mutation of *Fancl* also results in limited PGC populations in the embryonic gonad, similar to that seen in *Btbd12^βGeoFlox/βGeoFlox^* male embryos [65]. Interestingly, however, the residual PGCs can repopulate the testis gradually in the postnatal mouse such that, by 12 weeks of age, fertility is restored [65]. Thus, while the initial PGC defect may be similar in *Btbd12^βGeoFlox/βGeoFlox^* and *Fancl^−/−^* male embryos [65], the outcomes in terms of adult fertility are very distinct. In contrast to the age-related increase in fertility in *Fancl* nullizygous mice, and also to *Fanca* nullizygous mice, which show declining fertility with age [60], we see limited fertility in the *Btbd12^βGeoFlox/βGeoFlox^* males from 7 weeks of age onwards, with no subsequent restoration. Thus, it appears that mouse mutants for the FA complementation groups, while all showing similar anemia phenotypes, represent a spectrum of defects with respect to germ cell migration, proliferation, and differentiation. These differences underscore the importance of this family in genome stabilization within the germ line at all points in their development.

The pre-meiotic phenotype we observe in *Btbd12^βGeoFlox/βGeoFlox^* males is also temporally similar to that seen in *Ercc1* nullizygous animals [66] in that both mutations result in significant loss of germ cells prior to entry into meiosis. Indeed, the testicular phenotype of *Ercc1^−/−^* males at day 3 pp is remarkably similar, if not identical, to that presented herein [66]. In the case of *Ercc1*, however, no restoration of fertility with age has been reported for nullizygous animals and, in fact, the mice have reduced numbers of epididymal spermatozoa throughout reproductive life, all of which show distinct morphological defects [66]. The limited spermatozoa observed in *Btbd12^βGeoFlox/βGeoFlox^* males appear to be morphologically normal and capable of fertilization, and could be a result of the fact that these mice retain residual BTBD12 protein.

Beyond spermatogonial stages, we present evidence of a role for BTBD12 in prophase I progression in the mouse, congruent with the localization of BTBD12 protein on synapsed meiotic chromosome cores. The early localization of BTBD12 at these sites implies an early function in recombination events. Accordingly, in the *Btbd12^βGeoFlox/βGeoFlox^* animals, there is persistence in the signal for γH2AX across the autosomes beyond pachynema in the absence of BTBD12. While some 10% of cells show synapsis errors in *Btbd12^βGeoFlox/βGeoFlox^* spermatocytes, synapsis is largely unaffected in these mutants, which is in contrast to that seen for *FancD2* mutants in which spermatocytes show high levels of asynapsis in a subset of spermatocytes [59]. These data suggest a failure to repair DSBs in a timely fashion, leading to the persistent γH2AX and RAD51 observed at pachynema. However, we cannot rule out the possibility that the additional γH2AX signal is associated with persistent DNA damage arising during pre-meiotic replication events. Indeed, we did observe, by TUNEL labeling, a small fraction of spermatogonia undergoing apoptosis prior to meiotic entry, and so it is possible that if excessive DNA damage persists in the absence of BTBD12, then a proportion of these cells could avoid apoptosis and enter prophase I. Such a possibility can only be addressed through the use of prophase I-specific conditional knock-out strategies. Importantly, however, regardless of the timing of DNA damage induction (pre-meiotic or meiotic), these cells do not succumb to the pachytene checkpoint, as would be predicted from other knockout studies of early prophase I genes, including *Msh4/Msh5* and *Dmc1* [53], [54], [67]. Moreover, we do not see an overt increase in localization of TOPBP1, nor of MSH4 and MSH5 (data not shown), suggesting that the persistent DSBs observed at pachynema arise out of SPO11-induced events in early prophase I, and not as a result of DNA repair errors prior to meiosis.

Studies by the Sekelsky group were the first to indicate a role for vertebrate SLX4 orthologs in meiosis. These authors showed that the *Drosophila* Slx4 ortholog, MUS312, is essential for ensuring genomic stability and interacts with the MEI-9(XPF)/ERCC1 nuclease to produce meiotic COs [9], [14], [68], [69]. Thus *mus312* mutant flies exhibit >90% reduction in meiotic crossovers [14], but this is unrelated to Mus81 events since *Drosphila* Mus81 does not participate in CO regulation during meiosis [70]. Similarly, Saito *et al* describe a role for the *C. elegans* Slx4 ortholog, named HIM-18, in meiotic recombination, since *him-18* mutants exhibit reductions in crossing over of the order of 30–50%, depending on the chromosomal context [13]. Importantly, while *Mus-81* does not appear to play a critical role during meiosis in worms, double *mus-81;him-18* mutants show a more severe reduction in crossing over than *him-18* alone, suggesting co-operative roles for MUS-81 and HIM-18 in meiotic recombination in *C. elegans*. It is interesting to note that a pre-meiotic function was also described for *him-18* mutants, analogous to our observations for mouse BTBD12.

**Figure 8:**
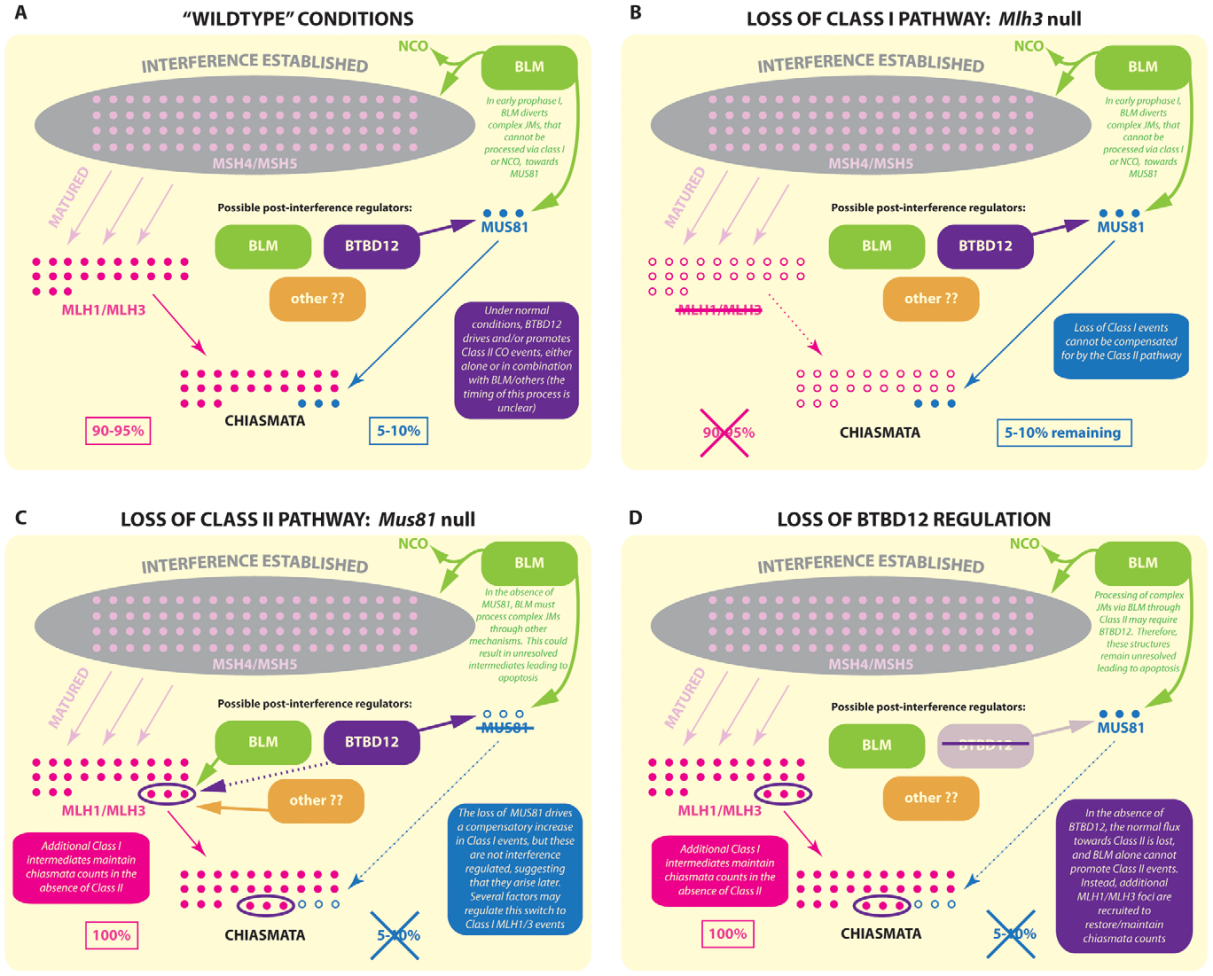
Model for BTBD12 involvement in meiotic crossover control in the Class I and Class II pathways. In wild type mice (A) MSH4/MSH5 foci (pale pink) accumulate on meiotic chromosomes during zygonema, and are subject to interference regulation (grey area). A subset of MSH4/MSH5 bound structures are then stabilized/matured by the accumulation of MLH1/MLH3 at pachynema, and these are designated as sites of Class I CO, which account for 90–95% of total crossovers. The remaining 5–10% of crossovers are generated by the Class II pathway, a major component of which is MUS81. It is thought that the precursors of these Class II COs may be aberrant JMs that, ordinarily, would be disassembled and/or processed towards NCO or Class I fates but that, by the complexity of their structure, avoid this fate. Thus, MUS81 processes those complex intermediates that “evade” BLM processing. (B) In the absence of MLH3, Class I COs cannot be generated, however a 5–10% subset of (presumably) Class II COs remain. These COs show CO breakpoints that are distal to the hotspot center and are more heterogeneic than MLH3 derived COs. (C) In the absence of MUS81, the full quota of Class I COs can be processed by MLH1/MLH3. However, the complex JMs that evade BLM processing cannot now be processed via Class II COs by MUS81. Instead, these structures are processed by some other mechanism or are left unresolved, increasing the apoptosis rate during prophase I. Meanwhile, however, through related or unrelated mechanisms, the Class I pathway upregulates acquisition of MLH1/MLH3 on MSH4/MSH5 sites, possibly taking advantage of the fact that there are excess numbers of the latter to which MLH1/MLH3 can bind and stabilize. Thus, the increase in MLH1/MLH3 foci by 5–10% does maintains the correct CO count. (D) The loss of BTBD12 strongly mirrors the loss of MUS81 in mammalian meiosis. An increase in Class I COs is indicated by a 5–10% increase in MLH1/MLH3 foci, whereas the final tally of COs in the form of chiasmata remains the same, indicating a loss of Class II events.

The role of BTBD12 in meiotic recombination is unclear at the current time, and it is interesting to note that the yeast ortholog, Slx4, does not appear to function in meiosis [71]. Given that BTBD12 interacts with components of both the Class I and Class II crossover pathways, as well as with BLM, it is well placed to play an important role in integrating CO decisions in mouse germ cells (Figure 8A). The loss of functional BTBD12 on meiotic chromosomes results in an increase in MLH1/MLH3 foci at mid-pachynema (Figure 5Q, 5R), similar to that seen in *Mus81* nullizygous males [28]. Also similar to the *Mus81* knockout mouse, we observe no increase in chiasmata at diakinesis in the *Btbd12^βGeoFlox/βGeoFlox^* males (Figure 6A, 6B). For *Mus81* nullizygous mice, we hypothesize that the additional MLH1/MLH3 foci can restore/maintain chiasmata numbers in the absence of a functional Class II pathway (Figure 8C). By contrast, loss of MLH1 or MLH3, the major Class I mediators at pachynema, cannot be compensated for by MUS81 or any other pathway (Figure 8B). Given the similarity of the *Btbd12* phenotype, described herein, to that of the *Mus81* nullizygous phenotype, our data suggest that BTBD12 may drive recombination intermediates towards Class II events, thereby promoting MUS81-mediated crossing over. In the absence of either MUS81 or BTBD12 (Figure 8D), however, CO numbers are maintained because of a compensatory increase of MLH1/MLH3 foci, possibly suggesting that BTBD12 does not mediate this switch between the two pathways, but can promote Class II pathway choices under certain conditions. In this regard, it is possible that BTBD12 acts in concert with BLM helicase.

Studies in yeast have suggested that the BLM ortholog, Sgs1, acts to limit the formation of aberrant JMs that arise from strand invasion events that involve both ends of the DSB (as just one example), instead producing substrates for the ZMM crossover pathway or instead resulting in a NCO fate, whilst Mus81-Mms4 can process those events that are not efficiently processed by Sgs1 even in wildtype situations [20], [21]. The absence of Sgs1, therefore, results in an overload of substrates for the Mus81 pathway [20], [21].

Given the model that emerges from the yeast data, together with the phenotypic characterization of *Btbd12^βGeoFlox/βGeoFlox^* mice described herein, we propose that the function of BTBD12 is to drive events towards Class II COs, possibly in a BLM-dependent fashion. This raises the question of the fate of those aberrant JMs that are not directed towards Class I or NCO pathways by the actions of BLM; those intermediates that, ordinarily, would be the substrates for MUS81 processing but which, in the absence of Class II-promoting BTBD12, still require resolution. Since it is unlikely that these aberrant structures are responsible for the additional MLH1/MLH3 sites that (presumably) maintain the normal chiasmata count, this suggests two important points. Firstly, the additional MLH1/MLH3 sites must be generated through some other mechanism, perhaps taking advantage of the fact that there is already an excess pool of MSH4/MSH5 foci from which to select for subsequent MLH1/MLH3-driven maturation/stabilization (see model Figure 8). Secondly, those BLM-processed JMs that fail to be diverted towards other recombination fates may remain un-repaired into late pachynema, as suggested by the persistent γH2AX and RAD51 on meiotic SCs in *Blm* conditional knockouts [72] and in *Btbd12^βGeoFlox/βGeoFlox^* males, and could well account for the gradual loss of spermatocytes via apoptosis leading up to the first meiotic division. Conflicting with these suggestions are our previous data showing increased chiasmata-like structures in the absence of *Blm*, that appear to be MLH1/MLH3-independent [72]. In addition, it should be noted that the complex intermediate structures reported for budding yeast have not yet been demonstrated in mammalian meiosis and, as such, our models, by necessity, relies on extrapolation from a number of organisms, most notably budding yeast.

BTBD12 and its orthologs have been shown to interact with a number of key players in the meiotic machinery, including Rad1-Rad10 [73], ERCC-1 [9], [14], [30], XPF (and its ortholog in *Drosophila*, MEI-9) [7], [9], [10], as well as with MUS81 and SLX1 [8], [10]. Studies by Svendson *et al* further demonstrated interactions between human BTBD12 and components of the DNA mismatch repair (MMR) family, including MSH2 [8]. Thus, a model emerges by which BTBD12 and its orthologs can mediate DNA repair and/or modification by directing the activities of certain nucleases in a substrate and context-specific manner. While the precise role of BTBD12 in mammalian meiosis remains to be further clarified, therefore, and its interactions with these various partners in mammalian germ cells have yet to be described, it is tempting to speculate on the function of BTBD12 based on localization data presented herein, on the phenotype of these mice, and based on previous studies of BTBD12 orthologs in other species. For example, studies *in vitro* have revealed distinct cutting activities for mammalian BTBD12 that are specific to its interactions with MUS81-EME1 (flaps and replication forks) and with SLX1 (HJ cleavage) [8], [10], [29]. Interestingly, the cleavage of HJs *in vitro* by BTBD12-SLX1 occurs in a near-symmetrical fashion, and in a manner similar to that seen for GEN1 [74], and it is plausible that such an activity may well confer at least limited HJ resolvase activity on the BTBD12-SLX1 complex in mammalian cells. This is in contrast to that seen for yeast Slx4, which cleaves HJ structures asymmetrically [2], [75] thereby reducing the likelihood of an *in vivo* resolvase role for the yeast protein. This difference in the cutting symmetry between the yeast and mammalian orthologs may explain why *slx4* mutants in yeast have no meiotic phenotype, while we observe a distinct meiotic phenotype in the *Btbd12^βGeoFlox/βGeoFlox^* mice.

BTBD12 was first identified as a candidate target of the ATM kinase [11] and, in line with this, Slx4 is a substrate of the yeast ATM/ATR orthologs, Mec1 and Tel1 [5]. The function of Slx4 in replication fork repair is dependent on this phosphorylation [55]. In line with this, our western blot analysis reveals a depletion of BTBD12 protein within the testis of adult and juvenile *Atm^−/−^* males compared to that seen in wildtype litter mates. BTBD12 protein is also lost from the chromosome cores of *Atm^−/−^* spermatocytes, while pre-meiotic spermatogonial proliferation appears to require both BTBD12 and ATM [64]. Thus, while it remains to be seen how ATM and BTBD12 co-ordinate their pre-meiotic functions in spermatogonia, we conclude that ATM activity is essential for the normal loading of BTBD12 onto meiotic chromosomes during prophase I and/or for the stabilization of BTBD12 at these sites. Interestingly, the increased loading of MLH1/MLH3 observed in *Btbd12^βGeoFlox/βGeoFlox^* spermatocytes mirrors the phenotype seen in *Spo11^+/−^Atm^−/−^* animals, the *Spo11* heterozygosity in this case rescuing the zygotene loss of spermatocytes in *Atm* single nulls and allowing progression to metaphase [31], [32], again pointing to a functional interaction between the ATM and BTBD12. *Spo11^+/−^Atm^−/−^* spermatocytes also show defects in the dynamics of sex chromosome synapsis and in the formation of the obligate crossover on the pseudoautosomal region (PAR) of the XY [31]. This phenotype is not shared with the *Btbd12^βGeoFlox/βGeoFlox^* males, suggesting that BTBD12 is not involved in this aspect of ATM function.

The *Btbd12^βGeoFlox/βGeoFlox^* strain of mice we have used herein do not, in all likelihood, represent a complete null allele, due to the presence of residual BTBD12 protein, as measured by both western blot and by immunohistochemistry. However, given the failure of this residual protein to accumulate on meiotic chromosome cores, at least to detectable levels, we consider this mouse to represent a significant impairment in BTBD12 function in germ cells. The use of the *Btbd12^βGeoFlox/βGeoFlox^* mouse, while not removing all of the endogenous protein, and while not permitting a focus on meiotic events alone, has been fortuitous in this instance, as it has highlighted the primordial germ cell proliferation defect, which otherwise would not have been evident using a meiosis-specific conditional null mouse. Clearly, any future meiosis-specific research should utilize a conditional approach, which would ensure BTBD12 absence only in meiotic cells, and allow much easier characterization of any defects as a result of loss of BTBD12 protein. Such studies are currently ongoing but will, by necessity, be lengthy.

As discussed, BTBD12 has many potential roles in DNA repair in mammals, from its function in somatic cell repair in human cell lines [7], [8], [10], [29], to its roles in pre-meiotic PGC proliferation and meiotic crossover formation demonstrated herein. The ability of BTBD12 to process not only HJs robustly, but other DNA structures, such as 3′ and 5′ flaps, as well [7], [8], [10], [29], indicates it has the potential to have numerous roles in DNA repair, both in mitosis and meiosis. Moreover, the evidence of functional interactions with a myriad of DNA repair proteins such as MUS81, BLM, XPF-ERCC1, MSH2-MSH3, and the Fanconi anemia genes, along with its well established binding partner SLX1, show that BTBD12 may integrate with several DNA repair complexes to effect its HJ (and other) processing abilities [7], [8], [10], [29]. These interactions no doubt contribute to the similarities between phenotypes at discrete stages of spermatogenesis for *Btbd12*, *Fancl*, *Ercc1*, and other mutant mouse models. Thus a clearer understanding of the function of mammalian BTBD12, both in the context of its multiple roles in gamete formation, and in its function in general genome stability/DNA repair will require more detailed knowledge of these key interactions.

## Materials and Methods

### Ethics statement

All animals used in this work were handled under strict guidelines imposed by Cornell Veterinary staff and by the Institutional Care and Use Committee (IACUC) under an approved protocol.

### Animals and genotyping

We obtained a line of *Btbd12* mice with a cassette inserted into the *Btbd12* gene, containing a β-galactosidase fused with a neomycin resistance gene, flanked by FRT sites, and LoxP sites flanking exon 3 of the *Btbd12* gene from the European Conditional Mouse Mutagenesis (EUCOMM) program (EPD0028_7_A08; *Btbd12^tm1a(EUCOMM)Wtsi^*). We termed these mice Btbd12*^βGeoFlox^*, to indicate that the FRT flanked and LoxP flanked regions are intact. Mice were genotyped using primers Btbd12_F 5′ CACTGAGCCATCTCACCAGC 3′ and Cas_R1 5′ TCGTGGTATCGTTATGCGCC 3′ to amplify the mutant allele (*Btbd12^βGeoFlox^* ^)^, and Btbd12_F 5′ CACTGAGCCATCTCACCAGC 3′ and Btbd12_R2b 5′ GGAGCCCAGTCTGGGACTCTG 3′ to amplify the wildtype allele (*Btbd12^+^*).

### Antibody preparation

The anti Rabbit polyclonal BTBD12 antibodies were against recombinant His-tagged murine BTBD12 peptide comprising amino acid residues 1–350 (“NT”) and 750–1100 (“CT”). For that, the corresponding cDNA fragment was cloned in pET-28 expression vector (Novagen) and recombinant proteins fused to a histidine tag were purified using Ni-NTA resin (Qiagen) following the manufacturer's instructions.

### Sperm counts

Epidiymides were removed from either *Btbd12^βGeoFlox/βGeoFlox^* or *Btbd12^+/+^* adult mice, placed in human tubule fluid (HTF) culture medium containing BSA (Specialty Media, Millipore), ripped open using micro forceps and the contents squeezed into the medium. The spermatozoa were cultured for 20 minutes at 32°C, then a 20 µl aliquot was removed and fixed in 480 µl 10% formalin. The fixed cells were gently mixed then intact spermatozoa counted using a hemocytometer.

### Histology

Testes were removed from pre-pubertal or adult mice and fixed either in Bouins fixative or 10% buffered formalin for 2–12 hours. Paraffin-embedded tissue was sectioned at 4 µm and processed for Hematoxylin and Eosin staining or immunohistochemical analyses using standard methods.

### Chromosome spread analysis

Testes were removed from adult *Btbd12^βGeoFlox/βGeoFlox^* or *Btbd12^+/+^* mice for the meiotic spread analysis, as well as for the focus counts, and processed as previously described [26]. Briefly, testes were removed and decapsulated into hypotonic sucrose extraction buffer (HEB, containing 1.7% sucrose) and left on ice for 60 minutes. Tubules were macerated on glass depression slides in a bubble of 0.03% sucrose and added to slides coated in 1% paraformaldehyde. The slides were dried slowly in a humidified chamber for 3 hours and washed in PBS containing Photoflo (Kodak, EMS). Ovaries were removed from day e19 female embryos and incubated in HEB for 20 minutes, before being macerated on a depression slide in 0.03% sucrose and added to a bubble of 1% PFA on a well slide, before drying as above.

### Immunofluorescence and immunohistochemistry

Slides were processed as described previously [76] using antibodies generated in this lab [26], generously donated by colleagues and available commercially. Immunohistochemistry was performed on formalin-fixed sections using rat monoclonal hybridoma supernatant against germ cell nuclear antigen-1 (GCNA-1), 10D9G11, for staining of germ cells [77], rabbit anti-BTBD12 antibody, rabbit anti-β-galactosidase or TUNEL staining (Chemicon) to detect cells undergoing apoptosis. γH2AX staining was described as either “normal” or “abnormal”, in both pachytene and diplotene cells from *Btbd12^βGeoFlox/βGeoFlox^* or *Btbd12^+/+^* spermatocytes. Abnormal cells were classified by >1 γH2AX focus per homologous chromosome core in pachynema, and 1 or more SC-associated γH2AX focus per nucleus in diplonema.

### Meiotic preparations of oocyte spindles

Oocyte spindles were prepared using a modification of techniques described previously [78], [79] and used subsequently in our laboratory [80]. Briefly, ovaries were removed by puncturing ovaries from unstimulated females at 24–26 days of age, and placed in Waymouth's media (GIBCO, Invitrogen Corporation, Carlsbad, CA) supplemented with 100 units of penicillin (base) and 10 µg of streptomycin (base)/ml, 10% fetal bovine serum, and 0.23 mmol/l sodium pyruvate. Primary oocytes at germinal vesicle (GV) stage were cultured in 20 µ*l* drops of KSOM (Millipore Corporation, Bedford, MA) overlaid with mineral oil (Chemicon, Millipore Corporation, Bedford, MA) and incubated at 37°C in an atmosphere of 5% CO_2_. After 2.5 hrs in culture, oocytes were transferred to fresh KSOM drops and scored for germinal vesicle break down (GVBD). In order to observe meiotic division at metaphase I, oocytes were cultured in KSOM for 8∼10 h and >18 h, respectively, and fixed in fibrin clots (below). Oocytes were fixed in fibrin clots, according to published methodology [78], prior to staining for β-tubulin (1∶500 mouse monoclonal antibody; Sigma-Aldrich, St. Louis, MO). Tubulin staining was visualized using a FITC-conjugated goat anti-mouse IgG (Jackson ImmunoResearch Laboratories, Inc., West Grove, PA), and counterstaining for DNA was achieved using 4′,6-Diamidino-2-phenylindole (DAPI).

### Statistical analyses

Testis weights, spermatozoa numbers, TUNEL analysis, immunofluorescent focus counts and diakinesis spread counts were all analyzed for statistical significance by using an unpaired t-test using Prism 4.0 software.

### Micronucleus assay material and methods

Analysis of micronucleus formation in peripheral blood cells was performed as previously described [35]. Briefly, peripheral blood was collected from the retro-orbital sinus, fixed in methanol, and incubated in bicarbonate buffer containing RNase A and anti-CD71: FITC antibody (Biodesign International). After washing and staining with propidium iodide, the cells were analyzed on a FACSCalibur flow cytometer (Becton-Dickinson, San Jose, CA).

## Supporting Information

Figure S1 *Btbd12^βGeoFlox/βGeoFlox^* mice show an increase in genomic instability. Micronucleus formation in wild type (white bar) and *Btbd12^βGeoFlox/βGeoFlox^* (grey bar) male mice (P<0.0001).
(TIF)

Figure S2 *Btbd12^βGeoFlox/βGeoFlox^* mice show an increase in gH2AX staining during late prophase I. gH2AX staining was quantified in wild type and *Btbd12^βGeoFlox/βGeoFlox^* pachytene and diplotene spermatocytes. Cells were classed as either normal (black bar) or abnormal (white bar) for gH2AX, and these numbers are represented as a % of the total number of cells counted (n = 209 and 212 for wild type and *Btbd12^βGeoFlox/βGeoFlox^*, respectively).
(TIF)

Table S1 Observed and Expected number of wildtype (*Btbd12^+/+^*), heterozygote (*Btbd12^+/βGeoFlox^*) and mutant (*Btbd12^βGeoFlox/βGeoFlox^*) animals born in the colony. Data obtained from 65 litters, for a total of 395 pups, and an average of 6 pups/litter.
(DOCX)

Table S2 Prospermatogonia numbers and apoptosis during neonatal development. Numbers indicate mean ± s.e.m. for GCNA1-positive and TUNEL-positive cells per seminiferous tubule, as assessed by immunostaining and TUNEL staining, respectively, of paraffin-embedded fixed sections from *Btbd12^+/+^* (wildtype), *Btbd12^+/βGeoFlox^* (heterozygote), and *Btbd12^βGeoFlox/βGeoFlox^* (mutant) males from embryonic day 16 (e16) through until day 3 post-partum (d3pp). Values were compared by Mann-Whitney U test and statistical values are provided below each age column. Number of tubules counted ranged from 10 to 43 from 1–3 mice.
(DOCX)

Table S3 Focus counts of recombination intermediates localized during prophase I in *Btbd12^+/+^* (WT), and *Btbd12^βGeoFlox/βGeoFlox^* (mutant) males spermatocytes. Numbers indicate mean ± s.e.m. for foci counted using antibodies against TOPBP1 in zygonema (, RAD51 in zygonema (zyg) and pachynema (pach), and CO markers MLH1 and MLH3, both in pachynema. Significantly different focus counts with a p value of <0.05 are indicated by the asterisks and were calculated using a standard unpaired t-test.
(DOCX)
