# Supplementary figures and images for: Mammalian BTBD12 (SLX4) Protects against Genomic Instability during Mammalian Spermatogenesis
Source: PLoS Genet. 2011 Jun 2;7(6):e1002094. doi: 10.1371/journal.pgen.1002094 (PMC3107204; doi:10.1371/journal.pgen.1002094)

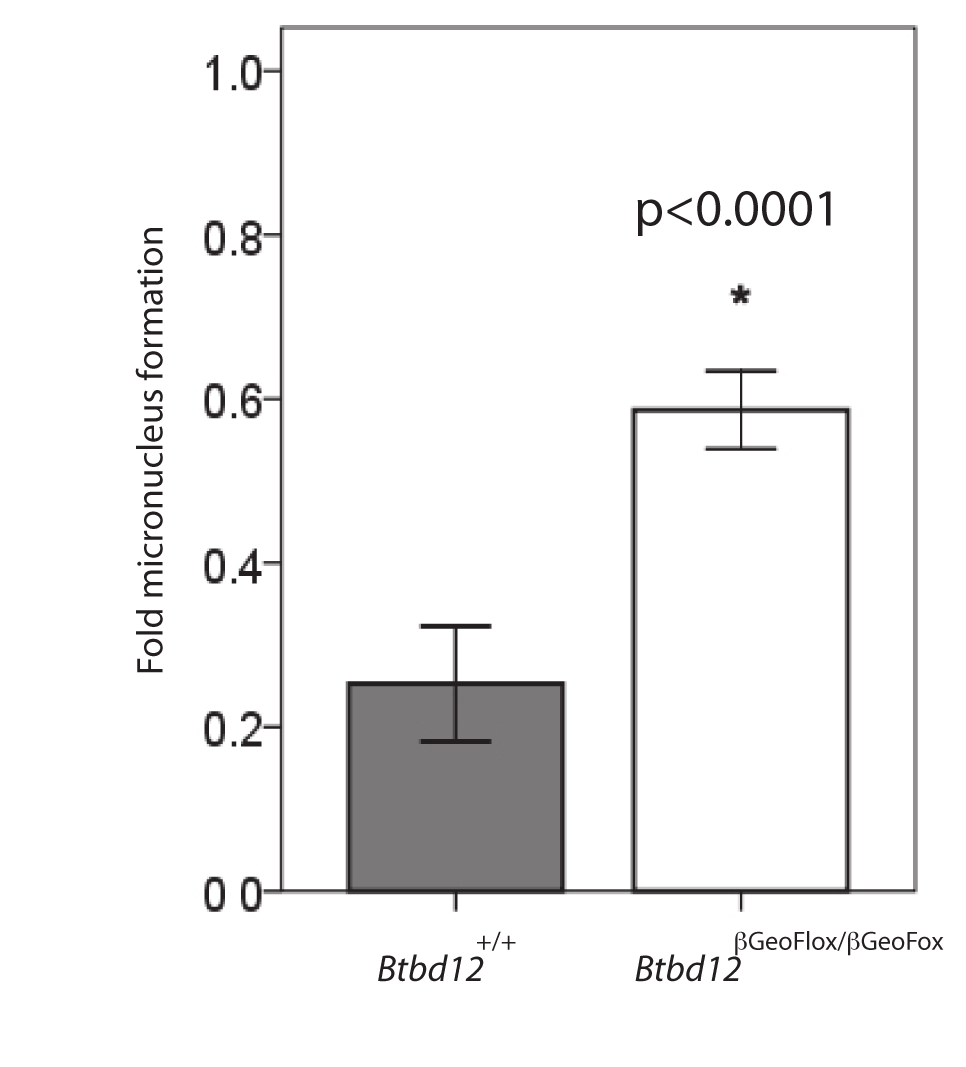

Supplement: Figure S1 — Btbd12βGeoFlox/βGeoFlox mice show an increase in genomic instability. Micronucleus formation in wild type (white bar) and Btbd12βGeoFlox/βGeoFlox (grey bar) male mice (P<0.0001). (TIF) [file pgen.1002094.s001.tif]

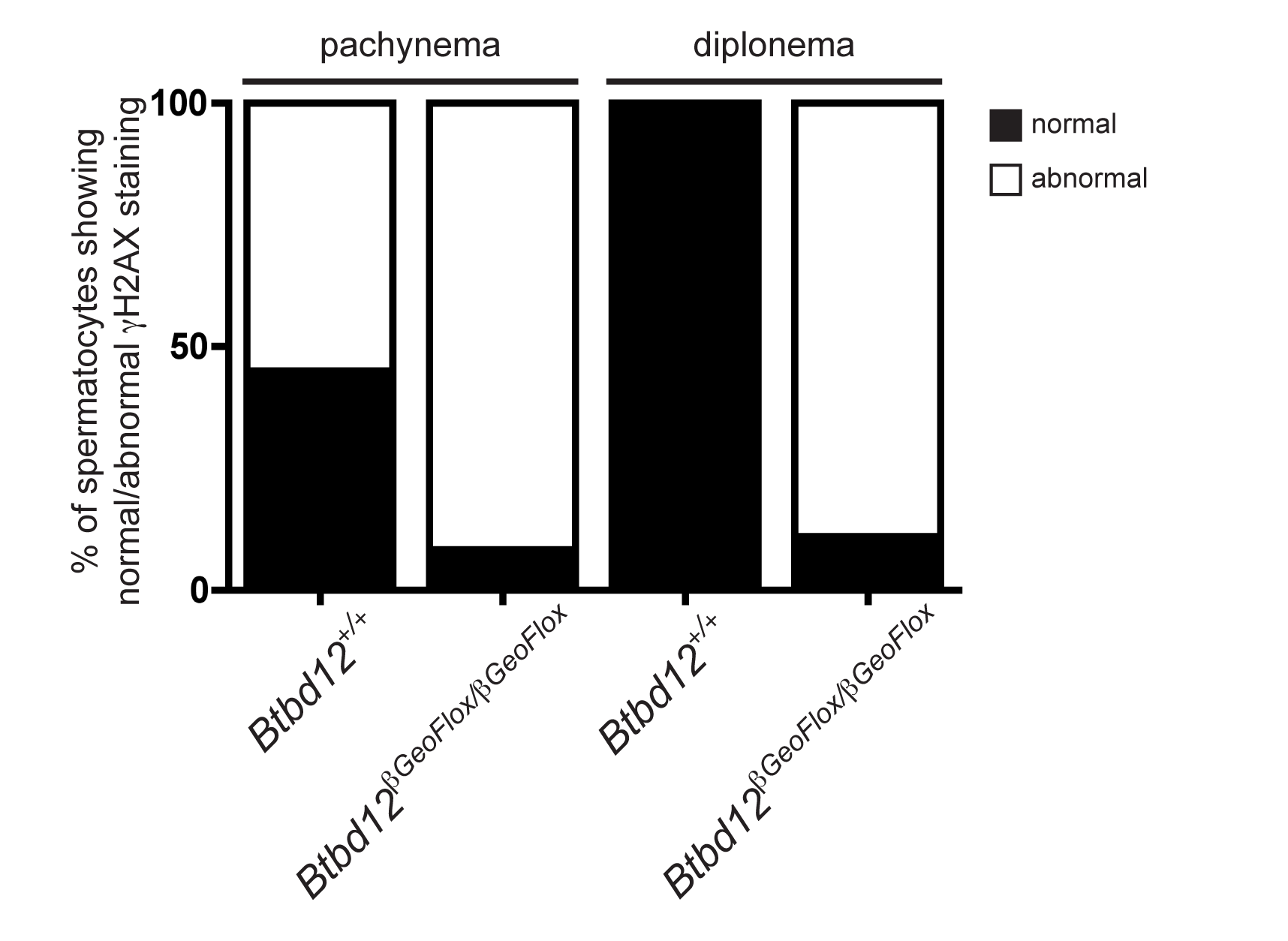

Supplement: Figure S2 — Btbd12βGeoFlox/βGeoFlox mice show an increase in gH2AX staining during late prophase I. gH2AX staining was quantified in wild type and Btbd12βGeoFlox/βGeoFlox pachytene and diplotene spermatocytes. Cells were classed as either normal (black bar) or abnormal (white bar) for gH2AX, and these numbers are represented as a % of the total number of cells counted (n = 209 and 212 for wild type and Btbd12βGeoFlox/βGeoFlox, respectively). (TIF) [file pgen.1002094.s002.tif]
