# Supplementary material for: Mammalian BTBD12 (SLX4) Protects against Genomic Instability during Mammalian Spermatogenesis
Source: PLoS Genet. 2011 Jun 2;7(6):e1002094. doi: 10.1371/journal.pgen.1002094 (PMC3107204; doi:10.1371/journal.pgen.1002094)
Supplement: Table S1 — Observed and Expected number of wildtype (Btbd12+/+), heterozygote (Btbd12+/βGeoFlox) and mutant (Btbd12βGeoFlox/βGeoFlox) animals born in the colony. Data obtained from 65 litters, for a total of 395 pups, and an average of 6 pups/litter. (DOCX) [file pgen.1002094.s003.docx]

|  | **Observed (n)** | **Expected (n)** | **% of total** |
| --- | --- | --- | --- |
| **Wildtype** | 119 | 98.75 | 30.1 |
| **Heterozygote** | 210 | 197.5 | 53.2 |
| **Mutant** | 66 | 98.75 | 16.7 |
| **Total** | 395 | 395 | 100 |
