# Supplementary material for: Mammalian BTBD12 (SLX4) Protects against Genomic Instability during Mammalian Spermatogenesis
Source: PLoS Genet. 2011 Jun 2;7(6):e1002094. doi: 10.1371/journal.pgen.1002094 (PMC3107204; doi:10.1371/journal.pgen.1002094)
Supplement: Table S2 — Prospermatogonia numbers and apoptosis during neonatal development. Numbers indicate mean ± s.e.m. for GCNA1-positive and TUNEL-positive cells per seminiferous tubule, as assessed by immunostaining and TUNEL staining, respectively, of paraffin-embedded fixed sections from Btbd12+/+ (wildtype), Btbd12+/βGeoFlox (heterozygote), and Btbd12βGeoFlox/βGeoFlox (mutant) males from embryonic day 16 (e16) through until day 3 post-partum (d3pp). Values were compared by Mann-Whitney U test and statistical values are provided below each age column. Number of tubules counted ranged from 10 to 43 from 1–3 mice. (DOCX) [file pgen.1002094.s004.docx]

|  | **GCNA1 positive cells** | | | **TUNEL positive cells** | | |
| --- | --- | --- | --- | --- | --- | --- |
|  | **e16** | **e18** | **d3pp** | **e16** | **e18** | **d3pp** |
| **Wildtype** | 8.58  + 1.23 | 8.39  + 0.80 | 2.08  + 0.19 | 0.21  + 0.096 | 0.19  + 0.10 | 0.32  + 0.21 |
| **Heterozygote** | 11.00  + 1.30 | 8.64  + 0.89 | 2.61  + 0.35 | 0.30  + 0.13 | 0.00  + 0.00 | 0.38  + 0.21 |
| **Mutant** | 8.75  + 1.09 | 2.10  + 0.60 | 0.92  + 0.28 | 2.42  + 0.90 | 1.88  + 0.81 | 0.79  + 0.22 |
|  |  |  |  |  |  |  |
| WT vs HET | n.s. | n.s. | n.s. | n.s. | ^a^ | n.s. |
| WT vs MUT | n.s. | p<0.0001 | p<0.0001 | p<0.05 | P<0.05 | n.s. |
| HET vs MUT | n.s. | p<0.0001 | p<0.0002 | p<0.05 | ^a^ | n.s. |

^a^comparisons with e18 heterozygote value are not possible with Mann-Whitney test, given that all the samples showed 0 TUNEL-postive cells.
