# Supplementary material for: Mammalian BTBD12 (SLX4) Protects against Genomic Instability during Mammalian Spermatogenesis
Source: PLoS Genet. 2011 Jun 2;7(6):e1002094. doi: 10.1371/journal.pgen.1002094 (PMC3107204; doi:10.1371/journal.pgen.1002094)
Supplement: Table S3 — Focus counts of recombination intermediates localized during prophase I in Btbd12+/+ (WT), and Btbd12βGeoFlox/βGeoFlox (mutant) males spermatocytes. Numbers indicate mean ± s.e.m. for foci counted using antibodies against TOPBP1 in zygonema (, RAD51 in zygonema (zyg) and pachynema (pach), and CO markers MLH1 and MLH3, both in pachynema. Significantly different focus counts with a p value of <0.05 are indicated by the asterisks and were calculated using a standard unpaired t-test. (DOCX) [file pgen.1002094.s005.docx]

|  | **WT mean** | **Mutant mean** | **P value** |
| --- | --- | --- | --- |
| **TOPBP1 (zyg)** | 176.2  + 31.82 | 166.2  + 19.37 | 0.39 |
| **RAD51 (zyg)** | 160.3  + 8.03 | 169.9  + 13.26 | 0.29 |
| **RAD51 (pach)** | 26.2  + 4.39 | 45.1  + 7.48 | 0.049* |
| **MLH1** | 23.28  + 0.41 | 25.56  + 0.61 | 0.0018* |
| **MLH3** | 24.62  + 0.28 | 25.57  + 0.38 | 0.0386* |
